# Scaffold-based 3D cell culture models in cancer research

**DOI:** 10.1186/s12929-024-00994-y

**Published:** 2024-01-14

**Authors:** Waad H. Abuwatfa, William G. Pitt, Ghaleb A. Husseini

**Affiliations:** 1 Materials Science and Engineering Ph.D. Program, College of Arts and Sciences, American University of Sharjah, P.O. Box. 26666, Sharjah, United Arab Emirates; 2 Department of Chemical and Biological Engineering, College of Engineering, American University of Sharjah, P.O. Box 26666, Sharjah, United Arab Emirates; 3 Department of Chemical Engineering, Brigham Young University, Provo, UT 84602 USA

**Keywords:** Three-dimensional (3D) cell culture, Scaffolds, Hydrogels, Decellularized tissues, Microfluidics, Extracellular matrix (ECM)

## Abstract

Three-dimensional (3D) cell cultures have emerged as valuable tools in cancer research, offering significant advantages over traditional two-dimensional (2D) cell culture systems. In 3D cell cultures, cancer cells are grown in an environment that more closely mimics the 3D architecture and complexity of in vivo tumors. This approach has revolutionized cancer research by providing a more accurate representation of the tumor microenvironment (TME) and enabling the study of tumor behavior and response to therapies in a more physiologically relevant context. One of the key benefits of 3D cell culture in cancer research is the ability to recapitulate the complex interactions between cancer cells and their surrounding stroma. Tumors consist not only of cancer cells but also various other cell types, including stromal cells, immune cells, and blood vessels. These models bridge traditional 2D cell cultures and animal models, offering a cost-effective, scalable, and ethical alternative for preclinical research. As the field advances, 3D cell cultures are poised to play a pivotal role in understanding cancer biology and accelerating the development of effective anticancer therapies. This review article highlights the key advantages of 3D cell cultures, progress in the most common scaffold-based culturing techniques, pertinent literature on their applications in cancer research, and the ongoing challenges.

**Graphical Abstract:**

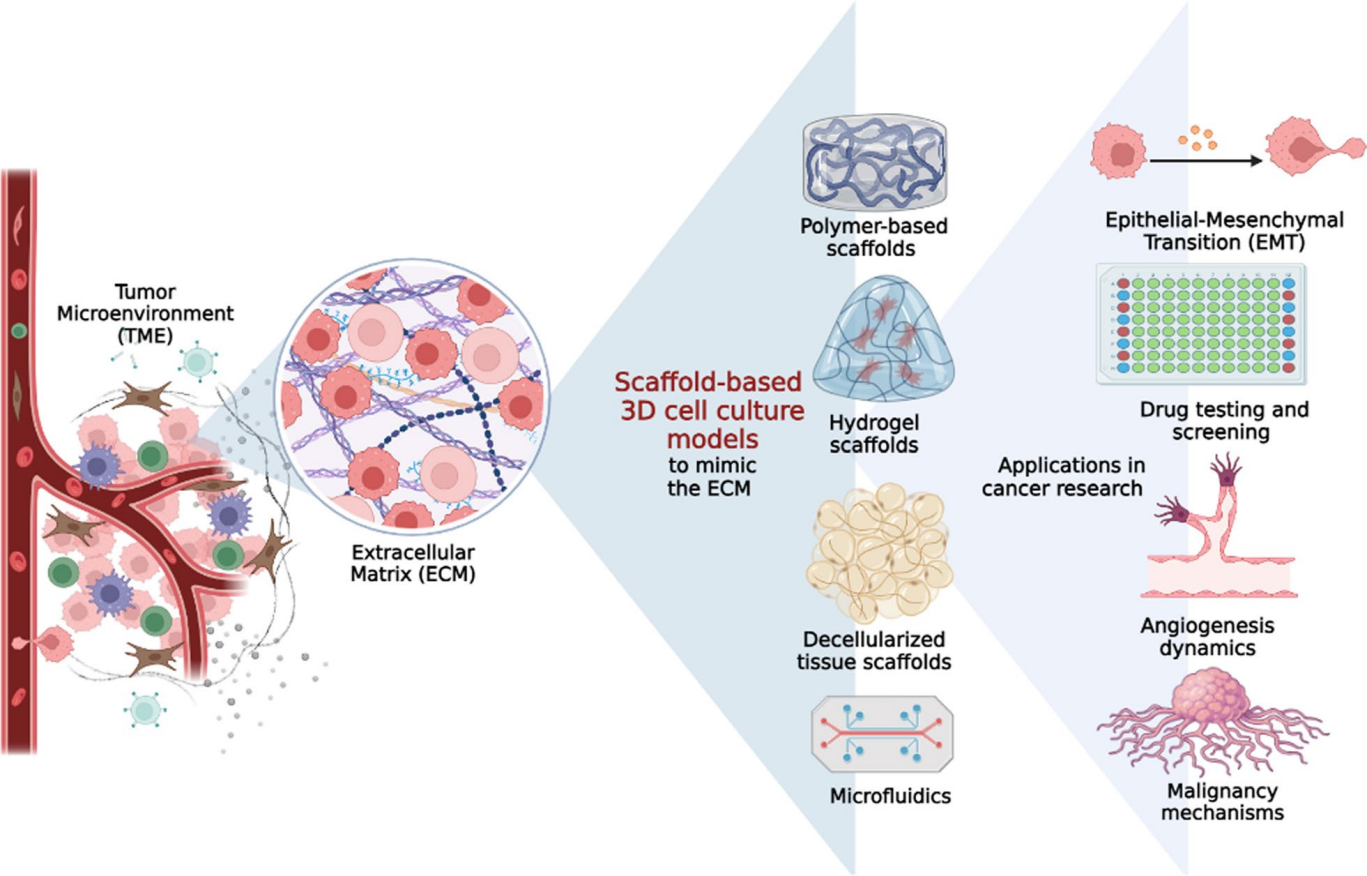

## Introduction

Cancer is a group of diseases characterized by the uncontrolled growth and spread of abnormal cells in the body. It is one of the leading causes of death worldwide, with millions of new cases and deaths each year [1]. Despite significant advances in cancer research and treatment over the years, the disease remains a major public health challenge and a substantial burden on patients, families, and society. Cancer research is crucial to develop new and effective treatments, improve patient outcomes, and find cures for this disease. As understanding of cancer biology and genetics continues to evolve, so do the approaches used to diagnose, treat, and prevent the disease. However, there is still much to learn about the complex mechanisms underlying cancer development and progression and the unique challenges posed by different types of cancers [2]. In addition, there is a need to develop more personalized and targeted therapies that can improve patient outcomes and minimize side effects. As such, cancer research must continue to innovate and advance to keep pace with the evolving understanding of the disease. This includes exploring new treatment modalities, developing more sophisticated diagnostic tools, and understanding the genetic and molecular mechanisms involved in its development and progression [3].

Two-dimensional (2D) cell culture is a commonly used technique to grow and maintain cells in the laboratory. Cancer research extensively uses it to study cells under controlled conditions, where they are grown on a flat surface supplied with a nutrient-rich liquid medium that provides the necessary nutrients for cell growth and survival. The growth medium used in cell culture varies depending on the type of cancer being studied and the desired goals of the study. One of the most critical aspects of cell culture for cancer research is maintaining cell viability and function, as cancer cells are highly susceptible to environmental changes [4]. Another challenge facing cell culture for cancer research is the ability to accurately model the complexity of human tumors. These are typically highly heterogeneous, comprising different cell types, including cancer, stromal, and immune cells. Understandably, 2D cell culture does not accurately mimic tumors’ three-dimensional (3D) environment [5]. The architecture and organization of cells in a 3D environment differ from those in a 2D environment, which can affect cell behavior and drug response. Recreating this complexity in a laboratory setting is difficult, as it requires the development of culture conditions that promote the growth and interaction of multiple cell types in a multifaceted environment [6]. Therefore, 3D cell culture models were developed as they offer sophisticated platforms that mirror the structural and functional complexities of in vivo tissues, providing valuable insights for cancer research and drug development. This review article highlights the key advantages of 3D cell cultures, the most common scaffold-based 3D culturing techniques, pertinent literature about applications in cancer research, and the challenges associated with these culturing techniques. Due to the topic’s vastness, this paper focuses on examining scaffold-based models of 3D cell cultures.

## Physiological relevance of 3D cell cultures to the ECM

Tumors are complex structures composed of cancer cells, non-cancerous cells (i.e., immune cells, fibroblasts, endothelial cells, etc.), and various extracellular matrix (ECM) components. The ECM plays a crucial role and contributes to the hallmarks of cancer in tumor progression, metastasis, and response to therapy [7, 8]. The ECM can (1) secrete growth factors and cytokines that promote cell proliferation and survival [9], (2) modulate the expression of genes involved in cell cycle regulation and apoptosis [10], (3) control the expression of telomerase, an enzyme that extends the telomeres of chromosomes, (4) secrete angiogenic factors that promote the formation of new blood vessels, thereby providing the tumor with the nutrients and oxygen it needs to grow [11], (5) promote the epithelial-to-mesenchymal transition (EMT), a process by which epithelial cells acquire the ability to migrate and invade other tissues [12], and (6) temper the immune response by influencing the recruitment and function of immune cells in the TME [13]. Romero-López and colleagues [14] tested how the ECM derived from normal and tumor tissues impacted blood vessels and tumor growth using reconstituted ECM. Tumor tissue obtained from liver metastases of colon tumors was subjected to hematoxylin and eosin (H&E) staining to confirm the successful decellularization of both colon and tumor tissues. Subsequently, significantly distinct protein composition and stiffness were observed among the reconstituted matrices, leading to notable variations in vascular network formation and tumor growth in both in vitro and in vivo. Fluorescence Lifetime Imaging Microscopy was employed to evaluate the free/bound ratios of the nicotinamide adenine dinucleotide (NADH) cofactor in tumor and endothelial cells to indicate cellular metabolic state. Notably, cells seeded in tumor ECM exhibited elevated levels of free NADH, indicating an increased glycolytic rate compared to those seeded in normal ECM. These findings underscore the substantial influence of ECM on cancer cell growth and the accompanying vasculature (e.g., increased vessel length, increased vascular heterogeneity). Alterations in the composition of tumor ECM, such as augmented deposition and crosslinking of collagen fibers, can be attributed to communication between tumor cells and tumor-associated stromal cells.

Every tissue type has a distinct ECM composition, topology, and organization [15]. These factors play a significant role in controlling cell function, behavior, and interactions with the microenvironment, as they generate spatial gradients of biochemicals and metabolites that, in turn, may elicit distinctive cell-mediated responses (e.g., differentiation, migration) [16]. Langhans [17] analyzed the chemical components of ECM and reported that it contains water, carbohydrates, and proteins, such as fibrous matrix proteins, glycoproteins, proteoglycans, glycosaminoglycans, growth factors, protease inhibitors, and proteolytic enzymes. Thus, ECM organization can influence cell genotypes and phenotypes, where such effects can be explored through 3D cell cultures [16, 18]. For example, variations in the gene and protein expression and activity of the epidermal growth factor receptors (EFGR), phosphorylated protein kinase B (phospho-AKT), and p42/44 mitogen-activated protein kinases (phospho-MAPK) in colorectal cancer cell lines (e.g., HT-29, CACO-2, DLD-1) affected the genotype and phenotype of cells in 3D cultures, as compared to 2D monolayers [19, 20]. Moreover, the ECM can influence cell morphology and expression of chemokine receptors. Kiss et al. [21] showed that 3D cultured prostate cancer cells (e.g., LNCaP, PC3) exhibited a high level of interaction between the cells and ECM, which resulted in the upregulation and overexpression of the CXCR7 and CXCR4 chemokine receptors. While 2D cell culture has been the mainstay of laboratory cancer research, it has become increasingly clear that this approach is inadequate in replicating the in vivo conditions that cells experience in the human body. As a result, researchers have been turning to 3D cell culture as a more physiologically relevant model for studying cellular processes and disease. A key advantage of 3D models for cancer research is that they can better mimic the complex microenvironment of tumors, including tumor morphology and topography, upregulation of pro-angiogenic proteins, dispersion of biological and chemical factors, cell–cell and cell–matrix interactions, gradients of oxygen and nutrients, and a more realistic ECM composition [6, 22, 23]. Necrotic, hypoxic, quiescent, apoptotic, and proliferative cells are often found in spheroid cell clusters at different phases of development [24]. Since the outer layer of the spheroid has greater exposure to the nutrient-supported medium, it contains a higher number of proliferating cells. Cells in the spheroid core are hypoxic and often quiescent as they receive less oxygen, growth agents, and nutrients from the media. This results in more physiologically relevant gradients in tissue composition that can better inform drug discovery and development [24]. Furthermore, 3D cell culture accurately depicts the cellular response to drugs and other therapeutic agents. Such a model’s spatial and physical characteristics influence the transmission of signals between cells, which alters gene expression and cell behavior [25]. Loessner et al. [26] demonstrated a flexible 3D culture method where a synthetic hydrogel matrix with crucial biomimetic properties provided a system for studying cell–matrix dynamics related to tumorigenesis. The 3D cultured cells overexpressed mRNA for receptors on their surface (e.g., protease, α3, α5, β1 integrins) compared to 2D cultured cells. Moreover, spheroid progression and proliferation depended on the cells’ ability to proteolytically transform their ECM and cell-integrin interactions. Consequently, the 3D spheroids showed higher survival rates in contrast to 2D monolayers after exposure to the chemotherapeutic agent paclitaxel, which indicates that it better stimulates in vivo chemosensitivity and pathophysiological events. Table 1 below summarizes studies using different 3D models to investigate different types of cancers.

**Table 1:** Summary of different 3D cell culture models used to study different types of cancer

Cancer type	3D culture model (cell type)
Lung cancer	- Multicellular tumor spheroids (Human lung cancer cell line SPC-A1 with the subpopulation of cancer stem-like cells) [27]. - Spheroids formed by the hanging drop method ( Colo699 and A549 cells) [28]. - A microfluidic system with soft hydrogel (A549 and HPAEpiCs cells) [29].
Glioblastoma	- Multicellular tumor spheroids (Glioblastoma tumor-initiating cells) [30]. - Suspension bioreactor (Glioblastoma cancer stem cells) [31].
Breast cancer	- Multicellular tumor spheroids (luminal stem cells) [32]. - Suspension bioreactor (breast cancer stem cells) [33]. - Hybrid system of biomimetic nano-cilia and microfluidics (MCF-7 cells) [34].
Pancreatic cancer	- Spheroids grown in a collagen matrix in a microfluidic device (BxPC-3, PANC-1, MIAPaCa-2 cells). - Spheroids formed using the hanging drop method (AsPC-1, BxPC-3, Capan-1, PANC-1, MIAPaCa-2, PSCs cells) [35]. - Polyacrylamide hydrogel (AsPC-1, BxPC-3, Suit2-007 cells) [36].
Ovarian cancer	- Multicellular spheroids grown in a microfluidic device (SKOV3 cells) [37]. - Multicellular spheroids ( OVCAR3 and SKOV3 cells) [38]. - Multicellular Tumor Spheroids (A1847, A2780, OVCAR3, OVCAR4, OVCAR5, OVCAR8, OVCAR10, PEO1, SKOV3 cells) [39].
Colon cancer	- Rotating Wall Vessel (HT-29 and HT-29KM cells) [40]. - Macroporous hydrogel scaffolds (HCT116 cells) [41]. - Spheroids grown in Matrigel (HCT116 cells) [42].

**Fig. 1:**
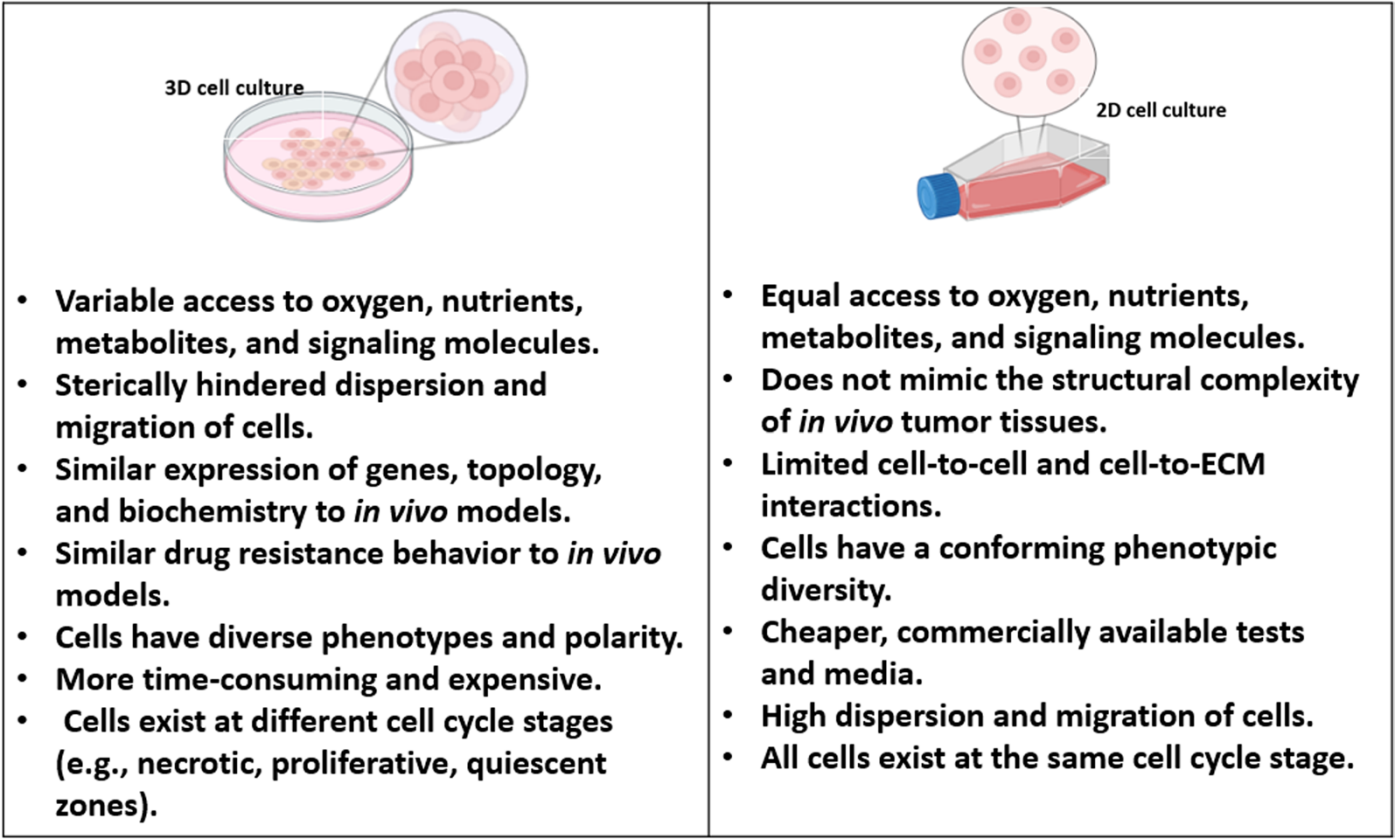
Characteristics of 2D and 3D cell cultures

Figure 1 summarizes the main characteristics of 2D and 3D cell cultures. The shift to 3D cell culture is a significant advancement in laboratory research, as it provides a more physiologically relevant model for studying cellular processes and disease. While some challenges remain to be addressed, the advantages of 3D culture outweigh the limitations of 2D culture. As technology continues to evolve, 3D culture is likely to become an increasingly crucial tool in cancer research and other fields of biomedical science.

Table 2 below provides a comprehensive overview of 2D, 3D, and other model systems employed in cancer research. Besides 2D and 3D cell cultures, tissues and organs present structural and functional intricacies, capturing organ-specific responses but posing challenges in maintenance and accessibility. Furthermore, model animals mimic in vivo systemic responses, yet ethical concerns, high costs, and species differences limit their utility. While clinically relevant, patient-derived samples present challenges in experimental control and sample heterogeneity [43]. It is noteworthy to highlight the difference between spheroids and organoids as both are commonly used terms within the scope of 3D cell cultures [44]. Organoids and spheroids are different 3D cell culture models that can be cultured with different techniques. Organoids, characterized by intricate structures replicating real organs or tissues, are composed of multiple cell types that self-organize to mirror tissue-like architecture, deriving from stem cells or tissue-specific progenitors. Due to their high biological relevance, they find applications in disease modeling, drug testing, and understanding organ development. Beyond organoids, tumoroids (i.e., tumor-like organoids), derived from patient cancer tissues containing tumor and stroma cells of the TME, are becoming advanced 3D culture platforms for personalized drug evaluation and development. In contrast, spheroids are simpler spherical cellular aggregates lacking the distinct organ-like structures of organoids. Comprising one or multiple cell types, spheroids are used to study fundamental cellular behaviors and drug responses in a 3D environment. While both contribute to 3D cell culture studies, organoids closely resemble real organs compared to the simpler cellular aggregates represented by spheroids [44]. Patient models are valuable tools that aim to replicate the complexities of human tumors, providing insights into disease mechanisms, therapeutic responses, and personalized treatment strategies. They can be utilized in Patient-Derived Xenografts (PDX), organoids and 3D cultures, patient-derived cell lines, liquid biopsies, and clinical trials [45].

**Table 2:** Commonly used models in cancer research and their advantages and disadvantages [45]

Model type	Features	Advantages	Disadvantages
2D cell culture	Involve cells grown in a flat, 2D layer, typically on culture dishes.	Well-established protocols. Rapid cell growth and division. Simple and cost-effective. Easy to manipulate and analyze. Suitable for high-throughput screening.	Oversimplified representation of in vivo conditions. Lacks cell–cell and cell–matrix interactions. Flat morphology, which may alter cellular responses.
3D cell culture	Represent a 3D arrangement of cells that mimics the spatial complexity of in vivo environments. These systems can be scaffold-free or scaffold-based, where cells are cultivated in scaffolds or matrices, allowing interactions that better replicate physiological conditions.	Enhanced drug response prediction. Allows for studying the TME facilitates investigation of tumor heterogeneity.	Variability in protocols and methodologies. Limited scalability for high-throughput assays.
Tissues and organs	Involve the cultivation of cells in configurations that mimic the structure and function of specific organs or anatomical parts. These systems provide a more complex and holistic environment than individual cells, allowing for a closer representation of in vivo conditions. By organizing cells into structures resembling organs or tissues, researchers can study interactions between different cell types and gain insights into the organ-specific responses to cancer and its treatments.	Preserve physiological cell functions. Enables interaction studies between cell types. Support long-term culture and functionality. facilitates drug metabolism studies. offer insights into organ-level responses. potential for personalized medicine approaches.	Challenges in standardization and reproducibility. Ethical consideration for human tissue use. Limited experimental control. Highly complex and challenging to replicate. Limited availability of organ models. Technical difficulties in maintaining viability.
Animals	Animal models, such as mice or rats, are living organisms used to study cancer.	Support evaluation of complex biological processes. Facilitate tumor growth, metastasis, and regression studies. Allow forin vivo evaluation of drug responses. Provide an intact immune system for immunotherapy studies. Allow for studying systemic effects of treatments.	Ethical concerns and regulatory challenges. Species-specific differences in drug metabolism. Costly, time-consuming and resource-intensive.
Patients	Patient model systems in cancer research involve using samples derived directly from patients. This can include patient-derived xenografts (PDX), organoids, or other personalized models. These systems aim to capture the unique characteristics of individual patients’ tumors, allowing for more tailored and patient-specific studies.	Addresses interpatient heterogeneity. Facilitates preclinical testing of patient-specific therapies. Enables personalized medicine approaches.	Challenges in obtaining patient samples. Limited availability of diverse patient cohorts. Difficulties in recapitulating the entire TME.

### Cell sources and 3D culture heterogeneity

In 3D cell culture, achieving an optimal balance between homogeneity and heterogeneity is intricately linked to the cellular source, considering stem cells, induced Pluripotent Stem Cells (iPSCs), or mixed primary cells derived from tissues [46]. Stem cells in in vitro cell culture encompass embryonic stem cells (ESCs), induced pluripotent stem cells (iPSCs), and adult or somatic stem cells. Embryonic stem cells exhibit high pluripotency, capable of differentiating into any cell type, but their use raises ethical concerns due to their origin from embryos. iPSCs are generated from somatic cells (e.g., skin or blood cells) through reprogramming, reverting them to an embryonic-like pluripotent state, but face reprogramming efficiency and potential tumorigenicity challenges. This transformation creates an extensive and diverse reservoir of human cells, capable of developing into any cell type required for therapeutic applications. Human-induced pluripotent Stem Cells (HiPSCs) are particularly relevant in cancer research (Table 3) [47]. Thus, the reprogramming process pioneered by Shinya Yamanaka has opened new avenues for advancing cancer biology, drug discovery, and regenerative medicine in cancer treatment. Lastly, adult or somatic stem cells are tissue-specific, mirroring the characteristics of their origin, and present fewer ethical concerns as they are derived from adult tissues. However, they have limited differentiation potential and a finite lifespan in culture. The selection of the cell source significantly influences the composition and behavior of the 3D culture. Stem cells and iPSCs, known for their pluripotency, introduce an inherent heterogeneity due to their ability to differentiate into various cell types [45, 46].

**Table 3:** Common sources of cells used in in vitro cultures and their merits and demerits

Cells used in in vitro cell culture	Merits	Demerits	Relevant studies
Stem cells	Maintain tissue-specific characteristics. Can be manipulated to exhibit disease-specific characteristics, offering a valuable tool for studying cancer in a controlled environment. Have the capacity for self-renewal, allowing for the production of daughter cells with similar properties.	Adult or somatic stem cells have a more restricted differentiation potential than pluripotent stem cells, limiting their versatility in modeling diverse tissues.	Myeloma stem cells [48]. Melanoma stem cells [49]. Breast cancer stem cells [50].
Induced Pluripotent Stem Cells (iPSCs)	The pluripotent nature of HiPSCs allows the creation of in vitro models that closely mirror the characteristics of cancer cells, providing valuable insights into cancer development and progression.	Inherent heterogeneity of the culture may affect the reproducibility and reliability of experimental results. Fully maturing iPSCs into specific cell types with desired functionalities can be challenging. In some cases, cells derived from iPSCs may not fully recapitulate the characteristics of their in vivo counterparts. iPSCs may exhibit genomic instability, impacting their differentiation potential and introducing variability in experimental outcomes. Using iPSCs, which involves reprogramming somatic cells, raises ethical considerations.	HiPSC-derived hepatocytes [51, 52]. HiPSC-derived cardiac myocytes [53, 54]. HiPSC-Derived gastric cells [55].
Primary cells	Biologically relevant, maintaining native cell characteristics. Reflect donor-specific variations. Useful for studying cell behavior, aging, and disease.	Limited lifespan in culture (senescence). Donor-dependent variability.	Primary breast cancer cells [56]. primary prostate cancer cells [57]. primary glioblastoma cells [58].

Furthermore, primary cells, derived directly from living organisms, possess unique characteristics that make them invaluable for in vitro studies. Maintaining biological relevance, these cells closely mimic the tissue or organ from which they are isolated, reflecting the intricacies of in vivo conditions. With donor-specific variability, primary cells allow researchers to explore genetic diversity’s impact on cell behavior, disease susceptibility, and drug responses. Retaining tissue-specific functions, differentiated primary cells are crucial for studying specific physiological processes and diseases associated with particular tissues [46]. However, these cells have challenges, including a limited lifespan and sensitivity to culture conditions. The finite replicative capacity and sensitivity contribute to the heterogeneity observed in 3D cell cultures, emphasizing the importance of carefully considering culture conditions and donor-specific variations to accurately represent in vivo scenarios. Despite these challenges, primary cells are vital in advancing our understanding of cell biology, disease mechanisms, and therapeutic development. Similarly, using mixed primary cells derived from tissues can contribute to a more heterogeneous cellular composition, resembling the complexity found in native tissues. Striking the right balance is crucial, as an excessive degree of heterogeneity may obscure specific responses, while too much homogeneity might oversimplify the representation of the tissue microenvironment. Therefore, a nuanced understanding of the cellular source is essential for tailoring 3D cell culture models to accurately reflect the intricacies of actual tissues and organs.

## Scaffold-based techniques for 3D cell culture

As explained above, developing 3D cell culture techniques that more accurately model the TME is a major area of focus in cancer research [6, 59]. Different approaches for 3D cell cultures exist and can be generally divided into scaffold-based and scaffold-free methods. Scaffold-free 3D cell culture refers to a cell culture technique in which cells are cultured and assembled into 3D structures without external scaffold material. Instead of being embedded within a supportive matrix, the cells self-assemble and interact with neighboring cells to form 3D tissue-like structures. Such cultures allow for more accurate cell–cell interactions, spatial organization, and physiological responses, making them valuable tools for various applications, including drug testing. They also usually have higher cell densities than scaffold-based models, which can influence cellular behavior, gene expression, and cellular functions. Lastly, non-scaffold models offer versatility and customizability in terms of cell types, culture conditions, and experimental designs. However, it is essential to consider that scaffold-free approaches might have limitations in providing mechanical support, shape control, and reproducibility compared to scaffold-based 3D cell culture methods [60]. As such, researchers often select the appropriate 3D cell culture method based on their specific research goals and the tissue or organ system they aim to model or engineer. Due to the topic’s vastness, the paper’s purviews' are limited to the examination of scaffold-based models of 3D cell cultures. Scaffolds are essential components in 3D cell culture systems, as they provide a 3D environment for cells to grow and interact with each other and their surroundings [61, 62]. Biomaterials employed in such models can be categorized into the following primary groups: polymer scaffolds, hydrogels, decellularized tissue scaffolds, and hybrid scaffolds (e.g., incorporating microfluidic devices). Tables 4 and 5 summarize the advantages and limitations of commonly used scaffold-free and scaffold-based 3D cell culture techniques, respectively.

**Table 4:** Description, advantages, and disadvantages of common scaffold-free 3D cell culture models

Non-scaffold-based technique	Description	Advantages	Limitations	References
Anchored spheroids or hanging-drop spheroids	They are also known as hanging-drop spheroids. Cells are suspended in a droplet of media that is hung from a surface. The droplet provides a 3D environment for cells to grow and form spheroids. Anchored spheroids do not require a scaffold or matrix to support the cells. Typically, they can be maintained for 10 to 14 days.	- Minimally invasive, and cells can be easily harvested without disrupting the 3D structure, allowing for downstream analysis. - Can achieve higher cell densities compared to other 3D cell culture models. - High-throughput screening.	- Not all cell types can form spheroids in hanging drops, limiting the range of cell lines that can be studied using this method. - Can be challenging to maintain a consistent shape and size of anchored spheroids, as gravity can cause the drops to merge or evaporate over time. - Limited scalability.	[63–67]
Non-adherent or ultra-low attachment spheroids	It is also known as a multicellular tumor sphere formation assay. Cells form tumor spheres through anchorage-independent growth mechanisms. Typically, these spheres can be maintained for up to 7 days.	- Inexpensive and replicable. - Cells show improved self-renewal capacity. - High-throughput screening of drugs and small molecules. - Particularly useful for characterizing stem cells. - Can be used for gene expression profiling.	- Labor-intensive. - Technically demanding. - Lack of complex tissue architecture. - May not accurately reflect in vivo conditions. - Insufficient nutrient and waste diffusion mechanisms. - Difficulty in controlling the size and shape of the structures. - Need specialized culture media. - Cellular aggregates might form due to cell mobility in media.	[68–71]
Scaffold-free bioprinting	Involves the direct printing of cell aggregates or individual cells without using an external scaffold. In this method, the cells are printed in a way that allows them to self-assemble and interact with neighboring cells, forming 3D tissue-like structures without the need for a supporting scaffold. Typically, they can be maintained for 2 to 4 days.	- Precise control over cell placement and scaffold architecture. - Can construct complex structures. - Bioinks are commercially available. - High-throughput screening.	- Still in the preliminary stages of development. - Expensive and requires expertise and high-tech equipment. - Difficult to upscale. - Cell viability depends on the technique used.	[72–77]
Magnetic levitation	It is a relatively new scaffold-free method for 3D cell culture. In this method, cells are mixed with magnetic nanoparticles and levitated using a magnetic field. As the cells levitate, they aggregate and form 3D structures. Magnetic levitation allows for the creation of complex 3D structures. Typically. The cell culture can be maintained for more than 7 days.	- Allows for precise control of cell–cell interactions. - The magnetic force and duration of exposure can be adjusted to control the size and shape of the spheroids or tissue constructs. - Non-invasive technique that does not require physical contact with the cells or tissues, minimizing potential damage.	- The use of magnetic nanoparticles may introduce potential toxicity concerns. - May not be suitable for all cell types as some cells may not respond well to magnetic fields. - Requires specialized equipment and technical expertise to set up.	[78–83]
Scaffold-free microfluidics	Fosters 3D cell culture environments without supporting matrices, such as hydrogels or scaffolds. These devices create controlled microenvironments within microfluidic channels to facilitate the aggregation and self-organization of cancer cells, promoting the formation of 3D structures, including spheroids or organoids.	- Elimination of expensive hydrogels or scaffolds reduces resource usage. - High-throughput screening to assess various conditions.	- Achieving consistent and reproducible results in scaffold-free cultures can be more challenging. - Certain cell types may require specific microenvironmental cues to form 3D structures. - Analyzing the behavior of cells in scaffold-free systems can be challenging and may require specialized techniques.	[84, 85]

**Table 5:** Description, advantages, and disadvantages of common scaffold-based 3D cell culture matrices

Scaffold type	Description	Advantages	Limitations	References
Natural polymers	They are derived from natural sources such as collagen, fibrin, or alginate. These scaffolds mimic the native ECM and provide a favorable microenvironment for cell growth.	- Biocompatibility: they closely resemble the ECM, promoting cell attachment, proliferation, and differentiation. - Bioactive properties: they can incorporate bioactive molecules that regulate cellular behavior and facilitate tissue regeneration. - Degradability: protein polymers will degrade over time, allowing for tissue remodeling and integration.	- Variability: natural polymers sourced from different origins can vary in composition and quality, leading to batch-to-batch variations. - Mechanical properties: they may have limited mechanical strength and stability, affecting their suitability for certain applications. - Immunogenicity: Some natural polymers can trigger immune responses in the body, potentially leading to adverse reactions.	[86, 87]
Synthetic degradable polymers	They are fabricated using synthetic biodegradable materials such as polycaprolactone (PCL) or poly(lactic-co-glycolic acid) (PLGA). These scaffolds offer precise control over their properties and can be tailored to specific requirements.	- Customizability: they allow for precise control over scaffold properties such as porosity, degradation rate, and mechanical strength. - Consistency: they offer consistent composition and quality, ensuring experiment reproducibility. - Stability: they can possess excellent mechanical stability and can withstand long-term cell culture conditions.	- Lack of bioactivity: they generally lack inherent bioactivity, requiring additional modifications or coatings to promote cell adhesion and functionality. - Biocompatibility concerns: some synthetic polymers may not be inherently biocompatible, necessitating surface modifications to enhance cell-material interactions. - Degradation byproducts: their degradation can generate acidic byproducts that may affect cell behavior and require careful consideration.	[88]
Hydrogels	Composed of a 3D network of hydrophilic polymer chains capable of retaining large amounts of water. They provide a hydrated and soft environment for cell growth.	- Biocompatibility: they offer a biocompatible environment similar to native tissues, facilitating cell viability and functionality. - Porosity and permeability: they possess porous structures that allow for nutrient and oxygen diffusion and waste removal. - Tunable properties: they can be easily modified to adjust mechanical properties, degradation rate, and incorporate bioactive molecules.	- Limited mechanical strength: they generally have low mechanical strength, limiting their use in load-bearing applications. - Swelling and stability issues: they can exhibit significant swelling and lack long-term stability, necessitating careful design and optimization. - Limited control over microstructure: achieving precise control over hydrogel microstructure and architecture can be challenging.	[89]
Decellularized tissues	It involves the removal of cellular components from native tissues, leaving behind the ECM structure and bioactive molecules. These scaffolds provide an environment that closely resembles the native tissue microenvironment.	- Native-like microenvironment: they retain the complex ECM composition and architecture, providing an environment that closely mimics native tissue. - Bioactive properties: the decellularized ECM contains bioactive molecules that can influence cellular behavior and support tissue regeneration. - Preserved tissue-specific characteristics: they retain tissue-specific characteristics such as mechanical properties, signaling cues, and growth factors.	- Limited availability: Obtaining sufficient quantities of decellularized tissues for large-scale experiments can be challenging. - Immunogenicity: Despite cellular removal, residual antigens or DNA fragments may still trigger immune responses in the recipient. - Complexity: they require meticulous preparation, involving multiple steps and specialized equipment, which may limit their widespread use.	[90]
Scaffold-based microfluidics	Cells are cultured within microscale channels that provide a controlled microenvironment for cell growth in a cell-laden scaffold.	- Can create precise spatial and temporal control over cell culture conditions (e.g., fluid flow, chemical gradients, and oxygen and nutrient delivery). - Can mimic tissue interfaces.	- Need for specialized equipment and expertise. - Limited scalability. - Can incorporate sensors and imaging tools that allow for real-time cell behavior and function monitoring. - Channel walls must be derivatized to be biomimetic.	[91, 92]

### Polymer-based scaffolds 

**Fig. 2:**
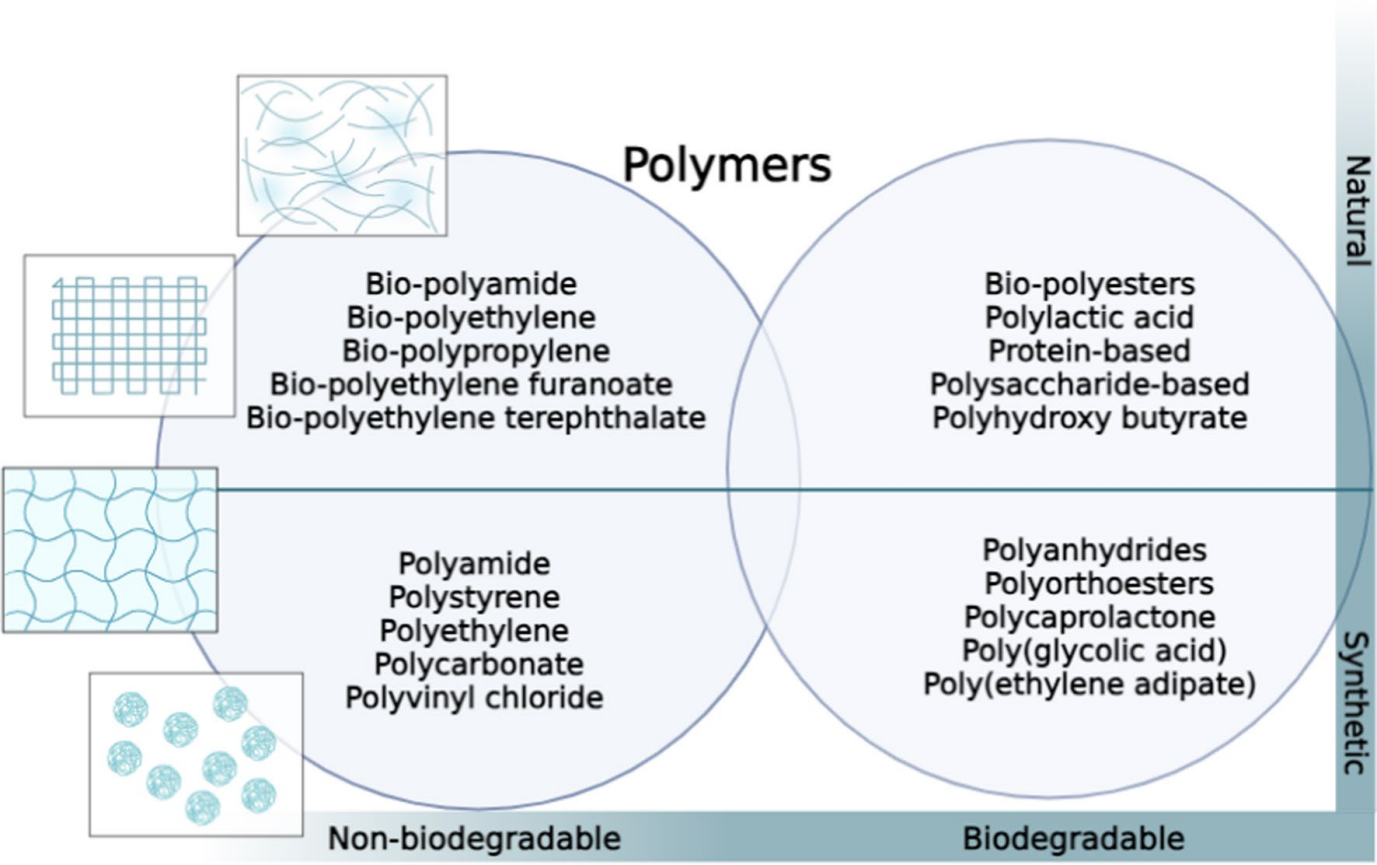
Classification of polymers used for fabricating polymer-based 3D cell culture scaffolds

Polymer scaffolds revolutionize 3D cell culture by providing a biomimetic environment imitating the natural ECM, fostering cell proliferation and differentiation, often with remarkable efficiency and precision. These scaffolds offer a versatile platform for studying complex cell behaviors and hold immense promise in cancer research applications. They can be generally classified as natural or synthetic-derived (see Fig. 2). Natural polymer scaffolds are made from naturally occurring polymers. They can be processed into various forms, including fibers, films, or porous structures. They can be further classified into two main categories: protein-based and polysaccharide-based scaffolds. Protein-based scaffolds are derived from large molecules composed of amino acids (e.g., collagen, silk, gelatin, fibronectin [93, 94]). Due to their bioactive properties, these scaffolds provide cell adhesion sites and can regulate cell behavior and tissue development. A 3D cell culture platform using collagen scaffolds was developed to investigate the tumorigenicity of cancer stem cells (CSCs) in breast cancer [95]. The study revealed that the 3D cell culture system demonstrated increased expression of pro-angiogenic growth factors, indicating a potential role in promoting blood vessel formation. Moreover, the overexpression of CSC markers such as OCT4A and SOX2, as well as breast cancer stem cell markers including SOX4 and JAG1, was observed in the 3D scaffolds, suggesting that the 3D model successfully replicated the molecular characteristics associated with CSCs. In terms of behavior, the 3D model more closely mimicked the characteristics of CSCs compared to an in vivo model, indicating its effectiveness in capturing the tumorigenic properties of CSCs. Therefore, the collagen scaffold-based 3D cell culture platform provided a valuable tool for studying CSC tumorigenicity in breast cancer, demonstrating the upregulation of pro-angiogenic growth factors, the overexpression of CSC and breast cancer stem cell markers, and a close resemblance to CSC behavior when compared to an in vivo model. Another study by McGrath et al. [96] used a 3D collagen matrix (GELFOAM™) to create an endosteal bone niche (EN) model, referred to as 3D-EN, for studying breast cancer cells’ quiescence and dormancy behaviors. The 3D-EN model effectively facilitated the identification of several genes associated with dormancy-reactivation processes, where among the tested cell lines, only MDA-MB-231 cells exhibited dormancy behavior, suggesting that they have a propensity for entering a dormant state in the simulated physiological conditions.

On the other hand, polysaccharide-based scaffolds are composed of long chains of sugar molecules (e.g., chitosan and hyaluronic acid). They are biocompatible, biodegradable, and can often be modified to adjust their physical and biological properties. Arya et al. [97] developed a 3D cell culture model using a chitosan scaffold, a natural polymer derived from chitin, to study breast cancer behavior. The scaffold was cross-linked with genipin, a natural cross-linker, to enhance its stability. The study found that the chitosan–gelatin (GC) scaffold provided a suitable environment for the growth of MCF-7 breast cancer cells, with the cells showing good adhesion and proliferation. The scaffold also supported the formation of cell clusters, which are more representative of in vivo tumor conditions compared to 2D cultures. The study concluded that the chitosan/gelatin scaffold could be useful for studying breast cancer in vitro, providing a more physiologically relevant model than traditional 2D cultures. GC scaffolds have been shown to support the formation of tumoroids that mimic tumors grown in vivo*,* making them an improved in vitro tumor model. These scaffolds have been successfully used to study lung cancer, as well as other types of cancer, such as breast, cervix, and bone [98]. These scaffolds have demonstrated gene-expression profiles similar to tumors grown in vivo, indicating their potential for studying cancer progression and drug screening for solid tumors [99]. The GC scaffolds have also been shown to improve the predictivity of preclinical studies and enhance the clinical translation of therapies [100]. Overall, the GC scaffolds provide a valuable tool for studying tumor development and evaluating the efficacy of anti-cancer drugs in an in vitro setting.

Synthetic polymer scaffolds (e.g., polylactic acid (PLA), polyglycolic acid (PGA), and polycaprolactone (PCL) can be tailored to have specific mechanical and biochemical properties. However, they can be less biocompatible than natural polymers and may require surface modifications to promote cell attachment and growth [60]. Palomeras et al. [101] tested the efficiency of 3D-printed PCL scaffolds for the culture of MCF7 breast cancer cells. The researchers found that the scaffold’s design, specifically the deposition angle, significantly influenced cell attachment and growth. Scaffolds with a deposition angle of 60° showed the highest cell counting after treatment with trypsin. Furthermore, the study found that the 3D culture in PCL scaffolds enriched the cancer stem cell (CSC) population compared to 2D culture control, increasing their Mammosphere Forming Index (MFI). The study concluded that 3D PCL scaffold culture could spur MCF7 cells to generate a cell population with CSC properties. This suggests its potential for studying CSC properties and screening new therapeutic agents targeting CSC populations. These efforts highlight the potential of natural polymer scaffolds in creating more physiologically relevant 3D cell culture models for cancer research. Using these scaffolds can enhance the understanding of cancer cell behavior and potentially lead to the discovery of more effective therapeutic strategies. Similarly, Rijal et al. [88] utilized modified gas foaming-based synthetic polymer scaffolds from poly(lactic-co-glycolic) acid (PLGA) and PCL for conducting 3D tissue cultures and animal models in breast cancer research. The research group investigated the response of MDA-MB-231 cells to anticancer drugs, their viability, morphology, proliferation, receptor expression, and ability to develop in vivo tumors using the 3D scaffolds. MDA-MB-231 cells were cultured on PLGA-coated 2D microscopic glass slides and in 3D-porous PLGA scaffolds to examine cancer cells’ survival on the polymeric substrata. The number of dead cells detected on the PLGA-coated glass slides and PLGA 3D scaffolds was negligible on Day 1. However, a significant increase in the number of dead cells was observed on the PLGA-coated glass slides compared to the 3D scaffolds on day 14. Additionally, the expression of ECM proteins and cell surface receptors on the synthetic polymers was investigated, where strong staining signals of type I collagen and integrin α2 were detected in both cell types using immunofluorescence (IF) microscopy. It is worth noting that integrin α2β1, which acts as a primary receptor for type I collagen, displayed a basal expression level in the 3D model. This expression pattern may promote breast cancer cell migration and tumor growth, as high levels of the integrin receptor tend to inhibit cancer cell migration. Notably, integrin α2 receptors showed a prominent colocalization with type I collagen, particularly around the cell edges, suggesting local deposition of type I collagen and subsequent binding of integrin α2 receptors, facilitating cell attachment and migration. Lastly, to evaluate the tumor formation capabilities of the polymeric porous scaffolds in mice, MDA-MB-231 cells were coated onto porous PLGA scaffolds and implanted into the mammary fat pads of NOD/SCID mice. Blank scaffolds without cells served as the negative control. As anticipated, the proliferating cell nuclear antigen biomarker Ki-67 was not detected in the blank scaffold implants. At the same time, its expression was significantly high within the tumors derived from the MDA-MB-231 cell-laden PLGA scaffolds. This finding suggested that the cancer cell population within the scaffolds exhibited rapid proliferation when embedded in the native breast tissues.

### Hydrogel scaffolds 

**Fig. 3:**
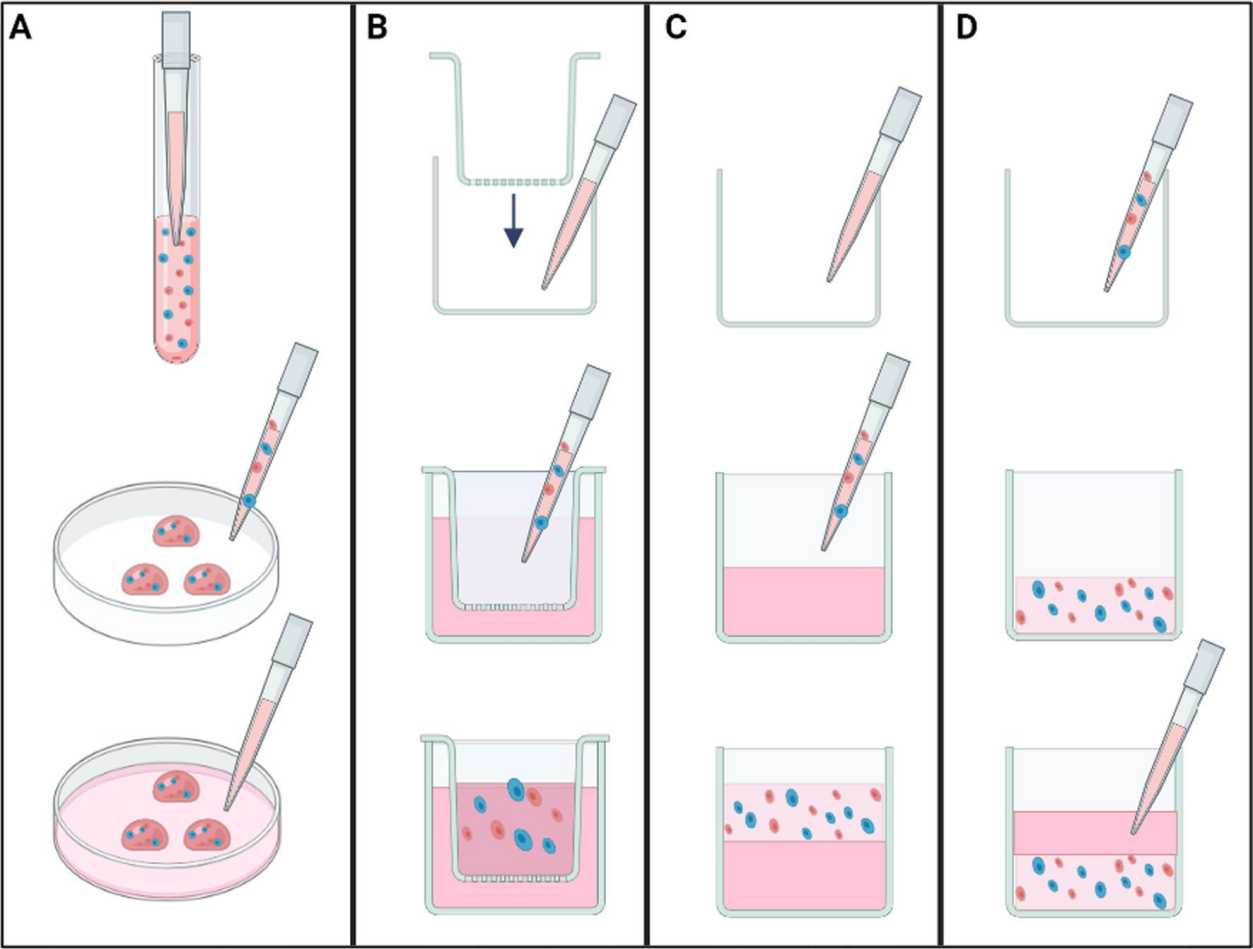
Common methods of hydrogel 3D cultures: **A** the dome technique: cells are mixed with temperature-sensitive hydrogels then seeded as droplets in the cell culture vessel, then carefully covered with media, **B** insert wells: media is added in the well whereas cell suspension (cell in hydrogel mix) is placed in the insert, then covered with another layer of media. Heterogeneous spheroids will form on the insert bottom, **C** gel-bottom support: the bottom of the well is covered with a thick layer of hydrogel, on top of which the cell suspension is placed, and **D** embedding technique: cells mixed with hydrogel are placed on the bottom and then covered with a layer of media to support spheroid growth in the matrix

Hydrogels are 3D networks of hydrophilic polymers (can be natural, synthetic, or hybrid), that can absorb large amounts of water or biological fluids while maintaining their structural integrity [102]. Figure 3 shows common techniques for culturing with hydrogel scaffolds. In the dome technique (see Fig. 3A), cells are mixed with temperature-sensitive hydrogels and then seeded as droplets within a cell culture vessel. This technique relies on careful temperature control to allow the hydrogel to polymerize and form a dome structure. Once the hydrogel has polymerized and the cell-hydrogel droplet is stabilized, it is delicately covered with cell culture media. This allows for a localized 3D cell culture in a larger vessel and can create multiple individual cell clusters or spheroids in a single plate. However, the maintenance of dome integrity can be challenging over time and might be affected by changes in temperature or physical disturbance. Also, it may not be suitable for long-term culture or cells requiring complex structural support due to the relatively simple and isolated 3D structure. Figure 3B illustrates the insert wells technique, which consists of porous inserts to hold the cell-hydrogel mixture while cell culture media is added to the well surrounding the insert. This separation creates a differential environment, allowing for nutrient exchange while maintaining a distinct 3D culture within the insert. Heterogeneous spheroids will eventually form on the insert bottom due to gravitational pull and cell–cell interactions. Such a model can be used to study cell invasion or migration by placing the cell-hydrogel mixture on one side of a permeable membrane and chemo-attractants on the other. The gel-bottom support method (see Fig. 3C) involves creating a thick layer of hydrogel at the bottom of a culture well, on top of which the cell suspension is placed. For instance, this method can be used for embedding cells within macroporous hydrogel scaffolds, such as AlgiMatrix^®^ (Thermo Fisher Scientific/Life Technologies, Carlsbad, USA)—an ionically gelled and dried scaffold that is conveniently provided in sterile pre-loaded disc format in standard cell culture well plates [103, 104]. To initiate the cell culture, a concentrated cell suspension in culture media is seeded on top of the hydrogel, where it is subsequently absorbed, resulting in the entrapment of the cells within the porous structure of the hydrogel. Lastly, in the embedding technique (see Fig. 3D), the cells are mixed with a hydrogel and directly placed at the bottom of a culture vessel, followed by a layer of culture media, allowing the cells to grow within the matrix of the hydrogel, thereby more accurately mimicking the in vivo 3D environment. This technique is beneficial for studying cell–cell and cell–matrix interactions, invasion, migration, and drug responses. However, it can be more technically challenging to embed cells evenly throughout the hydrogel; retrieving cells from the matrix for downstream analysis can be challenging. The permeability of the hydrogel to nutrients, gases, and wastes may need careful optimization to avoid creating a hypoxic environment or nutrient deprivation for cells located in the interior of the gel. Each of these methods must be selected based on the needs of the specific experiment and the type of cells being cultured. Additionally, the hydrogel composition and mechanical properties should be tuned according to the native ECM properties of the cell type of interest.

Due to their adjustable properties, synthetic hydrogels offer notable benefits in 3D cell culture. The RADA16-I peptide is a self-assembling peptide derived from a segment of Zuotin, a left-handed Z-DNA-binding protein originally discovered in yeast. This peptide has emerged as a novel nano-biomaterial due to its ability to form nanofiber scaffolds. Consequently, these scaffolds provide a supportive framework that promotes cell growth and fosters a conducive 3D milieu for cell culture. The peptide sequence can be modified to incorporate specific functional groups, thus fine-tuning the mechanical, chemical, and biological attributes of the resultant scaffold. This remarkable flexibility enables customization to align precisely with the unique demands of the cultured cells or the intended experimental objectives. These scaffolds, which are about 10 nm in diameter, are driven by positively and negatively charged residues through complementary ionic interactions. When dissolved in water, the RADA16-I peptide forms a stable hydrogel (nanofiber networks with pore sizes of about 5–200 nm) with extremely high water content at concentrations of 1–5 mg/mL, which closely mimics the porosity and gross structure of ECMs, making it suitable for the fabrication of artificial cell niches for applications in tumor biology. Yang and Zhao [105] prepared a RADA16-I peptide hydrogel that provided an elaborate 3D microenvironment for ovarian cancer cells in response to the surrounding topography. The 3D cell cultures exhibited a two to five-fold increase in drug resistance (paclitaxel, curcumin, and fluorouracil) compared to the 2D monolayers, which showed a good representation of the primary tumor and were likely to simulate the in vivo biological characteristics of ovarian cancer cells. Similarly, Song et al. [106] also proved that RADA16-I hydrogels can provide prominent and dynamic nanofiber frameworks to sustain robust cell growth and vitality. HO-8910PM cells, metastatic human ovarian cancer cells, were cultured in three hydrogel biomaterials, namely RADA16-I hydrogel, Matrigel, and collagen I. The specially designed RADA16-I peptide exhibited a well-defined nanofiber network structure within the hydrogel, providing a nanofiber-based cellular microenvironment similar to Matrigel and collagen I. Notably, the HO-8910PM cells exhibited distinctive growth patterns within the three matrices, including cell aggregates, colonies, clusters, strips, and multicellular tumor spheroids (MCTS). Moreover, the molecular expression of integrin β1, E-cadherin, and N-cadherin in 3D-cultured MCTS of HO-8910PM cells was elevated, and their chemosensitivity was reduced to cisplatin and paclitaxel in comparison to the 2D cell culture, evidenced by IC_50_ values and inhibition rates.

Furthermore, polyvalent hyaluronic acid (HA) hydrogels are considered synthetic, as they are typically created through chemical modification of HA molecules, introducing crosslinking agents or functional groups that enable the formation of a gel-like structure. This modification allows for control over the physical and mechanical properties of the hydrogel, such as its stiffness, degradation rate, and bioactivity. Suo et al. [107] engineered an ECM-mimicking hydrogel scaffold to replicate the native breast cancer microenvironment to provide an effective in vitro model for studying breast cancer progression. HA hydrogels from polyvalent HA derivatives were prepared through an innovative dual crosslinking process involving hydrazone and photo-crosslinking reactions. Hydrazone crosslinking is a versatile, reversible process that allows for rapid gelation, while photo-crosslinking stabilizes the formed hydrogel. Using this approach, they could efficiently produce HA hydrogels in under 120 s. It was found that the developed HA hydrogels closely resembled the topography and mechanical properties of breast tumors, and their characteristics (i.e., rigidity and porosity) could be fine-tuned by adjusting the amount of aldehyde-HA in the hydrogel formulation. This ability to modulate the mechanical properties of the hydrogels opens up possibilities for modeling different stages of tumor progression or different types of tumors. Moreover, a critical feature of the developed HA hydrogels was their dual-responsive degradation behavior, which was found to be responsive to glutathione and hyaluronidase. The glutathione responsiveness allows for degradation in response to the redox environment, which is often disturbed in cancer cells. Meanwhile, responsiveness of hyaluronidase makes the hydrogels sensitive to an enzyme that is typically upregulated in invasive cancer cells. Significantly, the HA hydrogel-cultured MCF-7 cells displayed upregulated expression of vascular endothelial growth factor (VEGF), interleukin-8 (IL-8), and basic fibroblast growth factor (bFGF) compared to their 2D cultured counterparts. These molecules are key mediators of angiogenesis and inflammation in cancer, suggesting that the HA hydrogel environment better replicates the conditions that promote these processes in tumors. Besides, the hydrogel-cultured cells exhibited enhanced migration and invasion abilities, which are key hallmarks of aggressive cancer cells. In vivo studies supported these results and confirmed the superior tumorigenic capacity of the MCF-7 cells cultured in HA hydrogels compared to those cultured in 2D. The outcomes of this research are anticipated to have far-reaching implications for both the in vitro study of breast cancer and the development of effective therapeutic strategies.

Another investigation by Wang et al. [108] supported that the level of methacrylation significantly influenced the hydrogel’s microstructure, mechanical characteristics, and capacity for liquid absorption and degradation. The refined hydrogel, synthesized through the photocrosslinking of methacrylated HA, displayed a highly porous structure, a high equilibrium swelling ratio, appropriate mechanical properties, and a degradation process responsive to hyaluronidase. It was found that the HA hydrogel promoted the growth and proliferation of MCF-7 cells, which formed aggregates within the hydrogel. In addition, 3D-cultured MCF-7 cells showed an increased expression of VEGF, bFGF, and interleukin-8, and enhanced invasion and tumorigenesis capabilities compared to their 2D-cultured counterparts. As such, the HA hydrogel has proven to be a dependable alternative for constructing tumor models. Gelatin methacryloyl (GelMA) is another commonly used natural biomaterial for 3D hydrogel scaffolds in cancer research. GelMA is derived from gelatin, a natural protein obtained from collagen-rich sources. It is modified by adding methacryloyl groups that enable it to undergo photocrosslinking when exposed to ultraviolet (UV) light. This property allows GelMA to form stable hydrogel networks, making it suitable for creating 3D scaffolds that mimic the tumor microenvironment (TME). The tunable mechanical and biochemical properties of GelMA hydrogels, biocompatibility, and ability to support cell growth make them valuable tools for studying cancer cell behavior, tumor invasion, drug screening, and other aspects of cancer research. Kim et al. [109] developed a 3D cell culture model for the bladder by employing a novel acellular matrix and bioreactor. GelMA was utilized as a 3D scaffold for the bladder cancer cell culture, with an optimal scaffold height of 0.08 mm and a crosslinking time of 120 s [110]. Subsequently, 5637 and T24 cells were cultured in 2D and 3D environments and subjected to rapamycin and Bacillus Calmette-Guérin (BCG) drug treatments. It was found that the 3D bladder cancer cell culture model exhibited a faster establishment process and greater stability when compared to the 2D model. Moreover, the 3D-cultured cancer cells demonstrated heightened drug resistance and reduced sensitivity compared to the 2D-cultured cells. Additionally, the researchers observed cell-to-cell interaction and basal activity in the 3D model, closely resembling the in vivo environment.

Along the same lines, Arya et al. [111] investigated the suitability of GelMA hydrogels as in vitro 3D culture systems for modeling key characteristics of metastatic progression in breast cancer, specifically invasiveness and chemo-responsiveness. The mechanical and morphological properties of the hydrogels were tuned by varying the percentage of GelMA used. Compression testing revealed that the stiffness of 10% GelMA hydrogels was within the range reported for breast tissue, making them suitable matrices for mimicking the breast viscoelasticity in vitro, as cells cultured on 10% GelMA hydrogels exhibited a higher proliferation rate compared to 15% GelMA in both cell lines tested, making them robust systems for long-term cell culture. Furthermore, proliferation studies showed that the GelMA hydrogels could sustain breast cancer cells longer than 2D cultures. Overexpression of genes associated with invasiveness was also observed in 3D cultured breast cancer cells, suggesting potential changes important for metastatic progression. The response to chemotherapeutic drugs was evaluated, and it was observed that 3D spheroids of breast cancer cells cultured on GelMA hydrogels exhibited decreased sensitivity to taxane drugs like paclitaxel. The study highlighted the importance of an adequate matrix pore size for cell penetration, migration, proliferation, exchanging oxygen, nutrients, and waste materials in and out of the 3D culture scaffolds. Significantly, these studies emphasized the importance of the 3D cancer cell culture model in establishing a patient-like model. Utilizing such models can achieve a more precise evaluation of drug responses, potentially leading to advancements in cancer treatment and other diseases.

Cells are known to respond to their mechanical environment in a process known as mechano-transduction, where they transmute mechanical stimuli into biochemical signals, subsequently prompting alterations in cellular behavior and functional operations. Curtis et al. [112] investigated the influence of mechanical stimuli on the cell proliferation, growth, and protein expression of 4T1 breast cancer cells, serving as a model for cells that metastasize to bone. The researchers used 4T1 breast cancer cells and implanted them in gelatin-mTGase hydrogels that mimicked the mechanical properties of bone marrow. The hydrogels had different moduli of either 1 or 2.7 kPa. The cells were cultured under different conditions, including static culture, perfusion of media through the hydrogel, and combined perfusion with cyclic mechanical compression for 1 h per day for 4 days. Control samples were cultured under free-swelling conditions. Immunostaining techniques were used to analyze the protein expression within the cell spheroids formed during the culture. The study found that mechanical stimuli significantly influenced the behavior of the 4T1 breast cancer cells. The cells formed spheroids during the culture period, with larger spheroids observed in statically cultured constructs than those exposed to perfusion or compression. In the stiffer gelatin, compressed constructs resulted in smaller spheroids compared to perfusion alone, while compression had no significant effect in the softer gelatin. The immunostaining revealed the expression of proteins associated with bone metastasis within the spheroids, including osteopontin, parathyroid hormone-related protein, and fibronectin. The proliferative marker Ki67 was present in all spheroids on day 4. The intensity of Ki67 staining varied depending on the culture conditions and gelatin stiffness. It highlighted the mechanical sensitivity of 4T1 breast cancer cells and demonstrated how mechanical stimuli can impact their proliferation and protein expression within soft materials that mimic the mechanical properties of bone marrow. The findings emphasized the role of the mechanical environment in the bone for both in vivo and in vitro models of cancer metastasis.

Understanding the influence of mechanical factors on cancer cell behavior is crucial for developing effective strategies to prevent and treat metastasis to bone, potentially leading to improved clinical outcomes for patients with advanced cancer. Similarly, Cavo et al. [113] investigated the impact of substrate elasticity on breast adenocarcinoma cell activity using mechanically tuned alginate hydrogels. The study evaluated the viability, proliferation rates, and cluster organization of MCF-7 breast cancer cells in 3D alginate hydrogels compared to standard 2D environments. The elastic moduli of the different alginate hydrogels were measured using atomic force microscopy (AFM). The results demonstrated that substrate stiffness directly influenced cell fate in 2D and 3D environments. In the 3D hydrogels with an elastic modulus of 150–200 kPa, the MCF-7 cells exhibited uninhibited proliferation, forming cell clusters with 100 μm and 300 μm diameters after 1 and 2 weeks, respectively. This unimpeded cell growth observed in softer hydrogels mimicked the initial stages of solid tumor pre-vascularization and growth. Furthermore, the multicellular, cluster-like conformation observed in the 3D hydrogels closely resembled the in vivo organization of solid tumors, demonstrating the advantage of 3D cancer models for replicating cell–cell and cell–matrix interactions. The study also highlighted the influence of microenvironment dimensionality on cellular morphology, as cells displayed a flat shape in 2D cultures while adopting a round shape in the 3D environment. Cell proliferation in the 3D setting depended highly on substrate stiffness, which impacted nutrient diffusion and intracellular signaling through a mechano-transduction mechanism. The findings underscore the importance of considering substrate stiffness in the design of 3D cancer models, as it directly affects cell viability, proliferation, and organization. By understanding the relationship between substrate stiffness and cellular behavior, researchers can develop more realistic in vitro models that better mimic the microenvironment of solid tumors. These models can advance our understanding of cancer development and aid in the development of targeted therapies by allowing for the investigation of cell–cell and cell–matrix interactions in a more accurate setting.

### Decellularized tissue scaffolds 

**Fig. 4:**
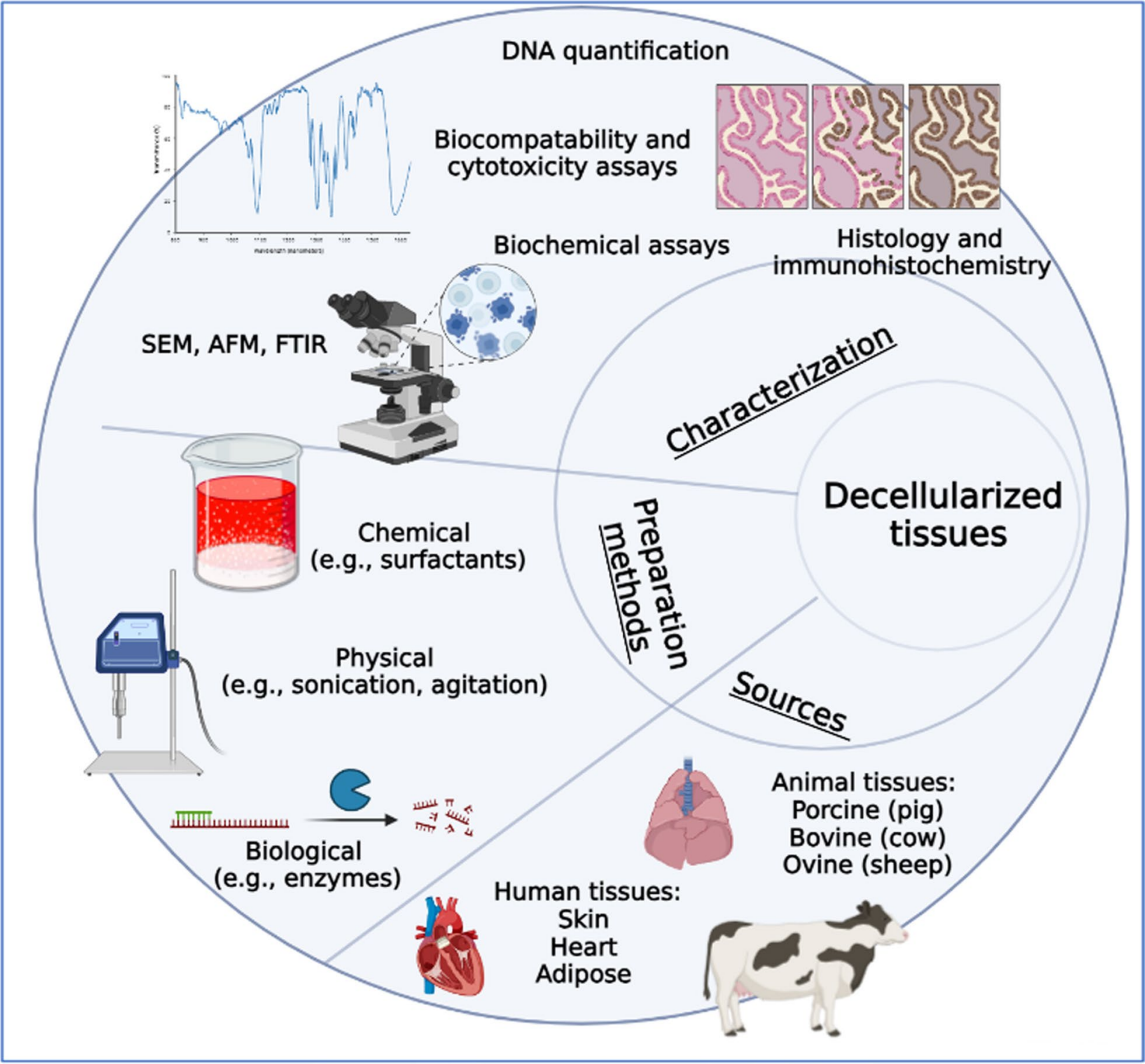
Preparation methods, characterization techniques, and sources of decellularized tissues used as scaffolds for 3D cell culture. SEM: scanning electron microscopy; AFM: atomic force microscopy; FTIR: Fourier-transform infrared spectroscopy

Decellularized tissues have had their cellular components removed, leaving behind the ECM. Decellularized tissues can be used as scaffolds for 3D cell culture, providing a natural environment for cells to grow and interact [114]. The use of decellularized tissues as 3D cell culture scaffolds offers several advantages. Firstly, they retain the intricate ECM composition, including structural proteins, growth factors, and signaling molecules, which play critical roles in cell behavior and tissue organization. This enables cancer cells to interact with the ECM more akin to in vivo conditions, influencing their adhesion, migration, invasion, and differentiation. Moreover, decellularized tissues offer spatial organization and architectural cues that guide cellular behavior. Preserving tissue-specific topography, such as vasculature, allows for studying angiogenesis and vascularization processes in cancer progression. These scaffolds also provide mechanical support and stiffness that influence cellular mechanotransduction, impacting cell morphology, proliferation, and gene expression patterns. They can be derived from various sources, including solid organs, such as the liver or lung, or specific tissue compartments, such as the ECM-rich decellularized basement membrane (see Fig. 4).

Landberg et al. [115] hypothesized that using a pre-clinical platform based on decellularized patient-derived scaffolds as growth substrates to account for hidden clinically relevant information and aid in modeling the individualized properties of microenvironments could be optimized for personalized treatment planning. Different decellularization techniques, such as chemical, physical, or enzymatic methods, remove cellular components while preserving the ECM integrity (see Table 6) [116]. The choice of decellularization method depends on the tissue type, desired scaffold characteristics, and the specific requirements of the study. Combinations of different techniques may also be employed to achieve optimal decellularization outcomes. However, challenges remain in the field. The immunogenicity and biocompatibility of decellularized tissues must be carefully considered to prevent adverse reactions when introducing foreign matrices into cell culture systems. Standardization and reproducibility of decellularization protocols are also crucial to ensure consistency across studies and facilitate comparison of results. Integration with advanced technologies, such as microfluidics or organ-on-a-chip systems, can further enhance the functionality and relevance of decellularized tissue models.

**Table 6:** Description, advantages, and disadvantages of decellularized tissue preparation techniques [116]

Method	Agents or techniques	Description	Advantages	Disadvantages
Chemical methods	Sodium dodecyl sulfate (SDS), Triton X-100, sodium deoxycholate.	Involve the use of various chemical agents which disrupt cell membranes and solubilize cellular proteins, facilitating the release of cellular components from tissues while preserving the ECM.	Relatively straightforward Efficiently remove cellular material.	The choice of chemical agents and their concentration must be carefully optimized to avoid damaging the ECM and compromising its structural and functional integrity.
Physical methods	Freeze–thaw cycles, agitation, sonication, hydrodynamic forces.	Involve mechanical forces or physical treatments to remove cellular components. For example, freeze–thaw cycles cause cell membranes to rupture and cellular contents to be released, while agitation methods utilize mechanical stirring or shaking to dislodge cells from the tissue surface.	Do not require chemical agents that may affect the ECM composition.	Can be challenging to completely remove all cellular remnants, including intracellular proteins and nucleic acids.
Biological methods	Nucleases (DNase, RNase) to degrade nucleic acids, proteases (such as trypsin or collagenase) to break down proteins, and glycosidases to remove glycosaminoglycans.	Utilize enzymes to selectively degrade cellular components, facilitating cell removal.	Offer selectivity in cellular component removal and can be tailored to specific tissues or ECM compositions.	Careful optimization of enzymatic concentrations, incubation times, and temperature is necessary to ensure efficient cell removal while preserving the integrity of the ECM.

D’Angelo et al. [117] developed a more representative 3D model of colorectal cancer liver metastasis using patient-derived scaffolds. These scaffolds, created by decellularizing tissue-specific ECM, retain the metastatic microenvironment’s biological properties and structural characteristics. The HT-29 CRC cell line was cultured within these scaffolds, obtained explicitly from cancer patients. The study observed increased cell proliferation and migration in the cancer-derived scaffolds, highlighting their ability to provide a more conducive environment for tumor cell growth and spreading. Furthermore, the 3D culture system demonstrated a reduced response to chemotherapy. HT-29 cells cultured in the cancer-specific 3D microenvironments showed decreased sensitivity to treatment with 5-fluorouracil and a combination of 5-fluorouracil with Irinotecan, when used at standard IC50 concentrations. The use of patient-derived scaffolds allows for the study of colorectal cancer metastasis progression and the assessment of their response to chemotherapy agents, to develop new therapeutic strategies and personalized treatments. Additionally, it provides an opportunity to identify potential prognostic biomarkers and therapeutic targets specific to peritoneal metastasis. Varinelli et al. [118] conducted a study that employed a tissue-engineered model for investigating peritoneal metastases (PM) in vitro, yielding similar conclusions. The model involved seeding PM-derived organoids onto decellularized extracellular matrices (dECMs) sourced from the peritoneum, enabling the exploration of intricate interactions between neoplastic cells and the ECM in the PM system. Both neoplastic peritoneum and corresponding normal peritoneum tissues were utilized to generate 3D-dECMs. Utilizing confocal reflection and polarized light microscopy techniques, the study observed disparities in tissue texture and the distribution and integrity of individual collagen fibers between normal and neoplastic-derived tissues obtained from three distinct PM patients. The results demonstrated that 3D-dECMs derived from neoplastic peritoneum exhibited a notably higher proportion of Ki-67-positive cells after 5 and 12 days. Furthermore, expression levels of specific genes critical for tissue architecture, stiffness, ECM remodeling, fibril generation, epithelial cell differentiation, resistance to compression, and regulation of angiogenesis were found to be elevated in 3D-dECMs generated from neoplastic tissue compared to those from normal tissue or Matrigel-based models. In summary, by utilizing patient-derived scaffolds and cutting-edge techniques, the researchers successfully developed more physiologically relevant models that significantly contribute to our comprehension of colorectal cancer and PM biology. These models, alongside others [119–122], offer valuable insights into the intricate interplay between tumor cells and the ECM, paving the way for the potential discovery of novel therapeutic targets and the development of personalized treatment strategies for peritoneal metastases.

Furthermore, decellularized tissue scaffolds provide an efficient platform to study the interactions between different components abundantly found in the ECM, like macrophages and endothelial cells. Macrophages and endothelial cells are known for their involvement in cancer progression in the context of the ECM within solid tumors, as they are often found in large numbers [123]. Macrophages within the tumor (often referred to as tumor-associated macrophages or TAMs) can be “hijacked” by cancer cells and reprogrammed to support tumor growth and progression. For example, they can promote cancer cell proliferation, enhance blood vessel formation (angiogenesis), assist in tissue remodeling, and suppress the immune response against the tumor. Pinto et al. [123] investigated how human colorectal tumor matrices influence macrophage polarization and their subsequent role in cancer cell invasion. To facilitate this, a novel 3D-organotypic model was utilized using decellularized tissues from surgical resections of colorectal cancer patients. This model preserved native tissue characteristics, including major ECM components, architecture, and mechanical properties, while removing DNA and other cellular components. The study found that macrophages within tumor matrices displayed an M2-like anti-inflammatory phenotype, characterized by higher expression of IL-10, TGF-β, and CCL18, and lower expression of CCR7 and TNF. Furthermore, it was observed that tumor ECM-educated macrophages effectively promoted cancer cell invasion through a mechanism involving CCL18, as demonstrated by Matrigel invasion assays. The high expression of CCL18 at the invasive front of human colorectal tumors correlates with advanced tumor staging, underscoring its clinical significance. The findings highlight the potential of using tumor-decellularized matrices as exceptional scaffolds for recreating complex microenvironments, thereby enabling a more comprehensive understanding of cancer progression mechanisms and therapeutic resistance.

Besides TAMs, endothelial cells express various adhesion molecules and chemokines, such as selectins, integrins, and members of the immunoglobulin superfamily, which can interact with ligands on cancer cells, facilitating their adhesion to the endothelial cell layer. This adhesion is a critical step in the extravasation process, where cancer cells exit the bloodstream and invade surrounding tissues to form metastases. Moreover, endothelial cells can signal and recruit macrophages and other immune cells to the tumor site. Once there, macrophages can be “educated” by the tumor to adopt a pro-tumor phenotype, suppressing the immune response and promoting tumor growth. Therefore, decellularized matrices are suitable for studying such interactions as they closely resemble the natural tumor environment, including native adhesion sites, signaling molecules, and mechanical cues. Helal-Neto et al. [124] examined the influence of dECM produced by a highly metastatic human melanoma cell line (MV3) on the activation of endothelial cells and their intracellular cell differentiation signaling pathways. The researchers studied the differences in the ultrastructural organization and composition of melanocyte-derived ECM (NGM-ECM) and melanoma-derived (MV3-ECM). Higher levels of tenascin-C and laminin and lower fibronectin expression were detected in MV3-ECM. Moreover, endothelial cells cultured in the MV3-ECM underwent morphological transformations and exhibited increased adhesion, mobility, growth, and tubulogenesis. The interaction between the endothelial cells and decellularized matrix induced integrin signaling activation, resulting in focal adhesion kinase (FAK) phosphorylation and its association with Src (a non-receptor tyrosine kinase protein). Src activation, in turn, stimulated the activation of vascular endothelial growth factor receptor 2 (VEGFR2), enhancing the receptor’s response to VEGF. The activation of VEGF and the association between FAK and Src was inhibited by blocking the αvβ3 integrin, which reduced tubulogenesis. In conclusion, the findings suggested that the interaction of endothelial cells with melanoma-ECM triggered integrin-dependent signaling, which led to the activation of the Src pathway that sequentially potentiated VEGFR2 activation and enhanced angiogenesis. Thus, progress in cancer biology relies on understanding the specific cellular responses influenced by the matrix signals within the ECM, as its nature inherently imposes spatial variations on cellular signaling, composition, topography, and biochemical factors. Table 7 summarizes some studies using hydrogel and decellularized tissue scaffolds for 3D cell cultures.

**Table 7:** Summary of studies using hydrogel matrices and decellularized tissue scaffolds in cancer research

Hydrogel (Origin)	Caner type/cell line(s)	Objective(s)	Findings	References
Agarose-gel (Natural)	Prostate cancer/ PC3, DU145	To compare the expression of the major EMT markers (i.e., E-cadherin, N-cadherin, α-smooth muscle actin (α-SMA), vimentin, Snail, Slug, Twist, and Zeb1) in 2D vs. 3D cell cultures.	- Significant morphological and phenotypical differences were observed between cells grown in 2D monolayers vs. 3D spheroids. - Low expression of mesenchymal phenotype markers in the 3D culture.	[125]
Collagen I (Natural)	Neuroblastoma/ SH-SY5Y	To compare growth mechanisms and gene expression of human neuroblastoma SH-SY5Y cells in 2D vs. 3D cultures.	- SH-SY5Y cells exhibited altered gene regulation, cell division, and neurite outgrowth behaviors in response to different sizes and compositions of the culture matrix. - 3D cultured cells displayed different gene expression and regulation of 1,766 genes, including those responsible for the ECM, cytoskeleton, and neurite outgrowth. - Further research is needed to determine how culture material characteristics (i.e., elasticity, permeability, surface energy, and chemical makeup) can influence relationships between cells and EMC.	[126]
Collagen I (Natural)	Ovarian cancer/ OV-2008	To recapitulate the architecture of the ECM in a solid tumor and investigate the motility and invasion capability of cells and their chemoresponsiveness.	- The 3D collagen models successfully recapitulated in vivo tumor-like microenvironment. - Collagen I stimulated invasion, EMT and drug resistance in ovarian cancer.	[127]
Matrigel (Natural)	Pancreatic cancer/ MCW670	To establish a patient-derived pancreatic cancer organoid co-culture 3D platform.	- The model allowed for accurate investigation of tumor-stroma and tumor-immune interactions in the organoid system. - Time-dependent activation of cancer fibroblasts was observed.	[128]
Matrigel (Natural)	Gastric cancer/ BGC-823	To evaluate the therapeutic effects of recombinant proteins (i.e., anti-EGFR and anti-EGFR-iRGD) in combination with chemotherapy (i.e., doxorubicin) on the drug uptake and efficacy in multicellular tumor spheroids.	- The combined approach enhanced drug penetration in the TME. - Further research is needed to investigate the underlying therapeutic mechanisms of the recombinant proteins with chemotherapy.	[129]
Matrigel (Natural)	Osteosarcoma/ MG-63	To investigate the effects of varying cellular arrangement in a 3D cell culture model.	- MG-63 spheroids encapsulated in hydrogel scaffolds exhibited higher invasion and drug resistance upon in vitro maturation. - 3D spheroids in hydrogel scaffolds led to increased invasion and drug resistance compared to randomly distributed osteosarcoma cells. - The findings indicated inherent physiological and drug response variances between 3D spheroids and cell-laden hydrogels.	[130]
Tasar silk fibroin (Natural)	Hepatocarcinoma/ HEPG2 and HepR21	To evaluate the effectiveness of the tasar silk fibroin scaffold as a 3D culture matrix for hepatic cancer cells.	- HepR21 cells grown in the 3D model compare favorably to HEPG2. cells in terms of increased adhesion, survival, metabolism, proliferation, and morphology. - The 3D model showed multicellular aggregations, indicative of tumor progression.	[131]
Cells-in-Gels-in-Paper (CiGiP) (Synthetic)	Lung cancer/ A549	To study the metabolic sensitivity of cells to ionizing radiation using a novel paper-based 3D model. The model created a decreasing gradient of oxygen and nutrients down the paper stacks, where the topmost cells were exposed to an oxygen-rich environment. In contrast, the bottom cells were exposed to oxygen-deficient conditions.	- Sensitivity to radiation declined with increasing cellular densities in single-layer cultures. - The model can be tuned to resemble tissue-like constructs by regulating oxygen exposure and nutrients supply.	[132]
Cell integrin-binding motifs (RGD peptides (Synthetic)	Ovarian cancer/ KLK4-7	To develop a 3D model that mimics cell proliferation, survival responses, and interactions with integrins in the TME.	- Results showed that combination therapy of paclitaxel with KLK/MAPK showed more pronounced effects than chemotherapy alone.	[133]
RADA16-I peptide (Synthetic)	Epithelial ovarian cancer/ A2780, A2780/DDP, and SK-OV-3	To evaluate the efficacy of the nanofiber scaffold as a 3D cell culture host and the effect of the material on cell adhesion, morphology, migration, and sensitivity to drugs.	- The RADA16-I peptide hydrogel scaffolds showed similar characteristics to collagen I scaffolds regarding cell adhesion and proliferative activity. - The 3D cell cultures exhibited a two to five-fold increase in drug resistance (paclitaxel, curcumin, and fluorouracil) compared to the 2D monolayers.	[105]

Decellularized tissue matrix	Caner type/ cell line(s)	Objective(s)	Findings	Refs
Small intestinal submucosa and mucosa	Lung cancer/ HCC827 and A549	To create a 3D model that allows for multiple read-out options, to monitor changes in cell signaling, proliferation, and apoptosis in response to drugs.	- The model was tested in silico and revealed that the cell lines represented lung carcinoma subgroups found in vivo. - Genetic variances were observed in the 3D model but not in the 2D model. - Results showed an increase in apoptosis and a decrease in viability upon administration of gefitinib.	[134]
Adipose tissue	Breast cancer/ MCF-7, SKBR3, BT474	To establish a 3D scaffold that resembles the breast cancer microenvironment.	- The cells grown in the 3D model showed growth and proliferation characteristics similar to in vivo xenografts. - The novel 3D platform bio-mimicked the in vivo microenvironment more accurately than existing Matrigel 3D cultures.	[135]
Rat adipose tissue	glioblastoma/ T98G; human hepatoma/ Hep3B; colon adenocarcinoma/ WiDr	to investigate the cellular behavior of various cancer cell lines in vitro using a platform derived from decellularized adipose tissue.	- The interactions between cells and the ECM varied depending on the proliferative capabilities of the tumor cells. - T98G and Hep3B cells were predominantly found at the edges of the matrix surface, likely due to their advantageous proximity to nutrient and oxygen sources. - The distribution pattern can be attributed to the tumor tissue’s location near capillaries, facilitating the active proliferation of cancer cells in that specific area. the proliferation potential of T98G and Hep3B cells remained evident throughout the third day of the experiment.	[136]
Colorectal cancer tissue and healthy colon mucosa	Colorectal cancer/ HT29 and HCT116 cells	To establish a patient-derived 3D preclinical model that can be used for drug evaluation in colorectal cancer.	- The 3D model demonstrated decreased sensitivity to 5-fluorouracil treatments compared to conventional 2D cultures. - The bioengineered 3D model holds promise as a reliable and patient-specific preclinical platform, effectively bridging the gap between in vitro and in vivo drug testing assays to facilitate more efficient cancer treatment strategies.	[137]
Healthy and cirrhotic liver tissues	hepatocellular carcinoma/ HCC cells	To examine and identify the distinct characteristics of the cirrhotic human liver ECM microenvironment, which promotes the development of hepatocellular carcinoma.	- Analysis of the decellularized 3D scaffolds revealed distinct proteins enriched in cirrhotic ECM compared to healthy ECM proteins. - Cell repopulation of the cirrhotic scaffolds resulted in the upregulation of genes associated with the transition from EMT and signaling pathways involving TGFβ. - Cirrhotic scaffolds showed higher concentrations of naturally occurring TGFβ1 than healthy scaffolds, indicating a unique TGFβ signaling environment. - Cells cultured in cirrhotic scaffolds exhibited significantly increased fibronectin secretion compared to cells in healthy scaffolds. - Stimulation with TGFβ1 led to the phosphorylation of canonical SMAD2/3 proteins, dependent on the specific ECM scaffold. - Treatment with the TGFβ-R1 kinase inhibitor Galunisertib effectively reduced TGFβ1-induced phosphorylation of SMAD2/3, regardless of the specific ECM scaffold.	[138]
Animal tongue tissue	tongue squamous cell carcinoma/ CAL27	To evaluate the feasibility of using decellularized tongue ECM for tongue squamous cell carcinoma research and tongue regeneration.	- A significant enrichment of integrin signaling in tongue ECM was observed. In addition to its impact on cell survival and proliferation, the tongue ECM also exerted coordinated effects on the interactions between cells and the ECM and neighboring cells, influencing the mode and extent of cell movement.	[139]
Porcine breast tissue	Breast cancer/ MCF-7 and hAMSCs	To establish decellularized breast scaffolds rich in glycosaminoglycans (GAGs) and collagen.	- The decellularized scaffolds facilitated the formation of cell clusters or spheroids, characterized by decreased expression of E-cadherin and elevated levels of tumor markers compared to 2D cultures. - The cell clusters or spheroids exhibited reduced chemo-sensitivity.	[140]

Decellularized tissue matrix	Caner type/cell line(s)	Objective(s)	Findings	References
Small intestinal submucosa and mucosa	Lung cancer/ HCC827 and A549	To create a 3D model that allows for multiple read-out options, to monitor changes in cell signaling, proliferation, and apoptosis in response to drugs.	- The model was tested in silico and revealed that the cell lines represented lung carcinoma subgroups found in vivo. - Genetic variances were observed in the 3D model but not in the 2D model. - Results showed an increase in apoptosis and a decrease in viability upon administration of gefitinib.	[134]
Adipose tissue	Breast cancer/ MCF-7, SKBR3, BT474	To establish a 3D scaffold that resembles the breast cancer microenvironment.	- The cells grown in the 3D model showed growth and proliferation characteristics similar to in vivo xenografts. - The novel 3D platform bio-mimicked the in vivo microenvironment more accurately than existing Matrigel 3D cultures.	[135]
Rat adipose tissue	glioblastoma/ T98G; human hepatoma/ Hep3B; colon adenocarcinoma/ WiDr	to investigate the cellular behavior of various cancer cell lines in vitro using a platform derived from decellularized adipose tissue.	- The interactions between cells and the ECM varied depending on the proliferative capabilities of the tumor cells. - T98G and Hep3B cells were predominantly found at the edges of the matrix surface, likely due to their advantageous proximity to nutrient and oxygen sources. - The distribution pattern can be attributed to the tumor tissue’s location near capillaries, facilitating the active proliferation of cancer cells in that specific area. the proliferation potential of T98G and Hep3B cells remained evident throughout the third day of the experiment.	[136]
Colorectal cancer tissue and healthy colon mucosa	Colorectal cancer/ HT29 and HCT116 cells	To establish a patient-derived 3D preclinical model that can be used for drug evaluation in colorectal cancer.	- The 3D model demonstrated decreased sensitivity to 5-fluorouracil treatments compared to conventional 2D cultures. - The bioengineered 3D model holds promise as a reliable and patient-specific preclinical platform, effectively bridging the gap between in vitro and in vivo drug testing assays to facilitate more efficient cancer treatment strategies.	[137]
Healthy and cirrhotic liver tissues	hepatocellular carcinoma/ HCC cells	To examine and identify the distinct characteristics of the cirrhotic human liver ECM microenvironment, which promotes the development of hepatocellular carcinoma.	- Analysis of the decellularized 3D scaffolds revealed distinct proteins enriched in cirrhotic ECM compared to healthy ECM proteins. - Cell repopulation of the cirrhotic scaffolds resulted in the upregulation of genes associated with the transition from EMT and signaling pathways involving TGFβ. - Cirrhotic scaffolds showed higher concentrations of naturally occurring TGFβ1 than healthy scaffolds, indicating a unique TGFβ signaling environment. - Cells cultured in cirrhotic scaffolds exhibited significantly increased fibronectin secretion compared to cells in healthy scaffolds. - Stimulation with TGFβ1 led to the phosphorylation of canonical SMAD2/3 proteins, dependent on the specific ECM scaffold. - Treatment with the TGFβ-R1 kinase inhibitor Galunisertib effectively reduced TGFβ1-induced phosphorylation of SMAD2/3, regardless of the specific ECM scaffold.	[138]
Animal tongue tissue	tongue squamous cell carcinoma/ CAL27	To evaluate the feasibility of using decellularized tongue ECM for tongue squamous cell carcinoma research and tongue regeneration.	- A significant enrichment of integrin signaling in tongue ECM was observed. In addition to its impact on cell survival and proliferation, the tongue ECM also exerted coordinated effects on the interactions between cells and the ECM and neighboring cells, influencing the mode and extent of cell movement.	[139]
Porcine breast tissue	Breast cancer/ MCF-7 and hAMSCs	To establish decellularized breast scaffolds rich in glycosaminoglycans (GAGs) and collagen.	- The decellularized scaffolds facilitated the formation of cell clusters or spheroids, characterized by decreased expression of E-cadherin and elevated levels of tumor markers compared to 2D cultures. - The cell clusters or spheroids exhibited reduced chemo-sensitivity.	[140]

### Hybrid scaffolds 

Integrating multiple scaffold types offers the potential to create 3D cell culture systems that closely mimic the physiological conditions of living tissues. This approach enables researchers to develop more accurate and biologically relevant models for studying cellular behavior, disease progression, and therapeutic responses. By combining different scaffold materials, such as natural and synthetic polymers or hydrogels, researchers can replicate the complexity and heterogeneity of the native tissue microenvironment. These hybrid scaffolds can provide a range of physical, chemical, and mechanical cues that influence cell behavior, including cell adhesion, migration, proliferation, and differentiation. Additionally, the combination of scaffolds can enhance the functionality of the 3D cell culture systems by incorporating specific features, such as the controlled release of growth factors or the inclusion of microvascular networks. Utilizing diverse scaffold types in 3D cell culture offers an innovative and promising approach for advancing our understanding of tissue biology, disease mechanisms, and developing more effective therapies. Bassi et al. [98] addressed the limitations of conventional therapies for osteosarcoma, a type of bone cancer, by introducing two innovative approaches in tumor engineering that aim to improve therapy outcomes. The study utilized hydroxyapatite-based scaffolds that mimic the in vivo TME, specifically emphasizing the CSC niche. Two types of scaffolds were employed: a biomimetic hybrid composite scaffold obtained through biomineralization, involving the direct nucleation of magnesium-doped hydroxyapatite (MgHA) on self-assembling collagen fibers (MgHA/Coll), and porous hydroxyapatite scaffolds (HA) produced by a direct foaming process. These scaffolds provided a framework for the subsequent investigation of the biological performance of human osteosarcoma cell lines (MG63 and SAOS-2) and enriched CSCs within these complex 3D cell culture models. Immunofluorescence and other characterization techniques were employed to evaluate the response of the osteosarcoma cell lines and CSCs to the biomimetic scaffolds. The results demonstrated the successful formation of sarcospheres, which are stable spheroids enriched with CSCs, with a minimum diameter of 50 µm. Comparing the advanced 3D cell culture models with conventional 2D culture systems, the study revealed the former’s superiority in mimicking the osteosarcoma stem cell niche and enhancing the predictivity of preclinical studies. The findings underscore the significance of the TME and emphasize the potential of combining CSCs with biomimetic scaffolds as a promising approach to developing novel therapeutic strategies for osteosarcoma. Further efforts can be focused on developing more sophisticated 3D models that accurately replicate the heterogeneity of the osteosarcoma microenvironment, incorporating patient-derived cells and elements such as immune cells and vasculature. Additionally, the advanced 3D cell culture models can serve as valuable tools for drug screening and personalized medicine approaches, further contributing to advancing osteosarcoma research and treatment strategies.

A unique cell culture technique known as “sequential culture” was used to establish a biomimetic bone microenvironment that facilitated the EMT of metastasized prostate cancer cells [141]. The approach involved incorporating bioactive factors from the osteogenic induction of human mesenchymal stem cells (MSCs) within porous 3D scaffolds, specifically polymer–clay composite (PCN) scaffolds, by incorporating hydroxyapatite (HAP) clay into PCL. The researchers also modified sodium clay Montmorillonite (Na-MMT) clay using 5-amino valeric acid to create HAPclay through in situ hydroxyapatite biomineralization into the intercalated nano clay. They performed RNA extraction and quantitative real-time polymerase chain reaction (qRT-PCR) analysis to investigate gene expression changes. Additionally, they conducted a comparative analysis of bone metastasis between the low and high metastatic cell lines, providing insights into their differential responses to the bone microenvironment. It was shown that both, the highly metastatic prostate cancer cell line PC-3 and the non-metastatic cell line MDAPCa2b, underwent MET transition when exposed to the biomimetic bone microenvironment in the 3D scaffold model. However, notable differences were observed in their morphological characteristics and cell–cell adhesion, suggesting distinct responses to the microenvironment. Additionally, quantitative variations in gene expression were observed between tumors generated using the two cell lines in the bone microenvironment. These findings are essential for developing targeted therapeutic strategies against prostate cancer bone metastasis. Bai et al. [142] conducted a study in which they incorporated graphene oxide (GO) onto a copolymer of polyacrylic acid-g-polylactic acid (PAA-g-PLLA) to create a stimuli-responsive scaffold. This scaffold, combined with PCL and gambogic acid (GA), exhibited a selective response towards tumors and demonstrated a significant accumulation of GO/GA in vitro breast tumor cells (MCF-7 cells) under acidic conditions (pH 6.8), while showing minimal impact on normal cells (MCF-10A cells) at physiological pH (pH 7.4). The study further revealed that the synergistic use of pH-responsive photo-thermal conversion was more effective in inhibiting tumor growth than independent treatments. In vivo experiments showed remarkable tumor suppression (99% reduction within 21 days) through tumor tissue disintegration, degeneration, and overall tumor suppression when treated with GO-GA scaffolds combined with photo-thermal therapy, in comparison to control groups or those treated with either GO-GA scaffolds or near-infrared (NIR) irradiation alone.

Microfluidics provide a versatile platform for 3D cell culture, offering both scaffold-based and scaffold-free approaches. Researchers can tailor the platform to suit the specific requirements of their experiments, whether involving cell-laden scaffolds or the aggregation of cells to form spheroids or organoids. The microfluidic setup allows for precise control over the microenvironment, including the flow of nutrients and oxygen, as well as the ability to introduce gradients of specific molecules. Lee et al. [143] utilized soft lithography to fabricate a 7-channel microchannel plate using poly-dimethylsiloxane (PDMS). Within separate channels, PANC-1 pancreatic cancer cells and pancreatic stellate cells (PSCs) were cultured within a collagen I matrix. The study observed the formation of 3D tumor spheroids by PANC-1 cells within five days. Intriguingly, the presence of co-cultured PSCs resulted in an increased number of spheroids, suggesting a potential influence of PSCs on tumor growth. In the co-culture setup, PSCs exhibited heightened expression of α-smooth muscle actin (α-SMA), a marker associated with fibroblast activation, as well as various EMT-related markers, including vimentin, transforming growth factor-beta (TGF-β), TIMP1, and IL-8. These findings indicated that PSCs may induce an EMT-like phenotype in PANC-1 cells, potentially promoting tumor invasiveness, chemoresistance, and metastasis. Upon treating the co-culture with gemcitabine, the survival of the spheroids did not exhibit significant changes. However, when combined with paclitaxel, the tumor spheroids demonstrated a notable inhibitory effect on growth. The model revealed a complex interplay between PANC-1 cells and PSCs within the TME. Nonetheless, the combination of gemcitabine and paclitaxel showed promise to overcome resistance and inhibit tumor growth. The implications of these findings are significant for understanding the complex interplay between tumor cells and the surrounding stromal cells within the TME. Tumor-stroma interactions play a critical role in cancer progression and therapy response. Using microfluidic-based 3D co-culture models allows researchers to better recapitulate the in vivo conditions, providing a more accurate representation of tumor behavior and therapeutic responses.

**Fig. 5:**
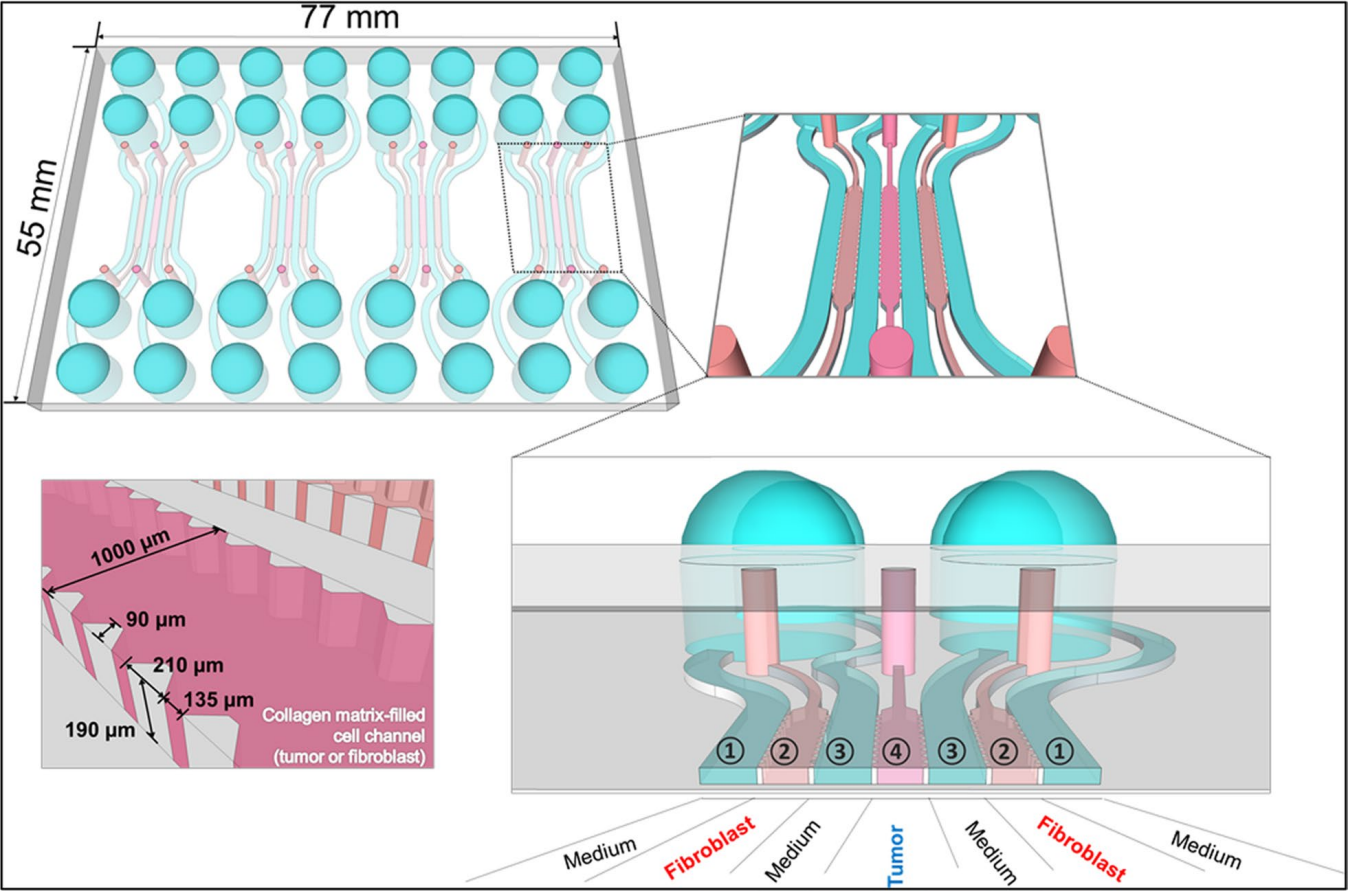
illustration of the microfluidic chip used in 3D co-culture of human colorectal cancer cells (HT-29) and normal colorectal fibroblasts (CCD-18Co) in a collagen matrix. The chip comprised 4 units, each featuring 7 channels for cell loading or media fill. Cancer and fibroblast cells were loaded into channels 4 and 2 in the co-culture, while channels 1 and 3 were designated for media fill. A cell loading channel’s detailed structure and dimensions are illustrated at the bottom left. Figure adapted from [145]

Likewise, Chen et al. [144] developed a microchannel plate-based co-culture model to recreate the in vivo TME by combining Hepa1-6 tumor spheroids with JS-1 stellate cells (liver cancer)—the novel model aimed to mimic key aspects of EMT and chemoresistance observed in tumors. The integration of these cell types in 3D concave microwells allowed for the formation of 3D tumor spheroids in 3 days. The experimental setup was optimized to ensure optimal culture proliferation conditions and appropriate interactions between Hepa1-6 and JS-1 cells. Co-cultured JS-1 cells displayed noticeable changes in cellular morphology, including an increase in the expression of α-SMA. In contrast, the co-cultured Hepa1-6 spheroids exhibited higher expression levels of TGF-β1 than those cultured alone. These findings suggested that JS-1 stellate cells induced an EMT-like phenotype in the Hepa1-6 cells, potentially contributing to increased invasiveness and resistance to chemotherapy. Jeong et al. [145] conducted a similar study involving the formation of 3D spheroids composed of human colorectal carcinoma cells (HT-29) using a microfluidic chip. They reported a notable enhancement in HT-29 growth when co-cultured with fibroblasts (see Fig. 5). This enhancement was demonstrated by a 1.5-fold increase in the percentage change in spheroid diameter over 5 days. Furthermore, after 6 days of culture, the co-cultured spheroids exhibited reduced expression of Ki-67, a marker associated with proliferation, while showing increased fibronectin expression. These findings indicated altered cellular behavior compared to the spheroid monocultures. The presence of fibroblasts in the co-culture environment also led to their activation, as evidenced by an upregulation in the expression of α-smooth muscle actin (α-SMA) and an increase in migratory activity. This reciprocal interaction between the spheroids and fibroblasts within a microfluidic chip established a dynamic relationship. Additionally, when exposed to paclitaxel, the co-culture displayed a survival advantage over 2D monoculture, suggesting the potential role of fibroblasts in conferring drug resistance. Integrating the 3D tumor spheres and CAFs within a collagen matrix-incorporated microfluidic chip provided a valuable tool for studying the TME and evaluating drug screening and efficacy. This approach allowed for the replication of essential interactions between tumor cells and stromal components, which are known to influence cancer progression and therapeutic response. By utilizing the proposed microfluidic chip-based model, researchers can delve into the intricate dynamics of the TME and explore novel therapeutic approaches. The ability to control and better mimic the in vivo conditions within the chip provides a valuable platform for investigating drug responses and evaluating the effectiveness of anticancer treatments. Further exploration and refinement of this model could lead to significant advancements in our understanding of tumor biology and the development of targeted therapies for improved patient outcomes. Table 8 summarizes some studies using microfluidic-based systems to develop 3D cell cultures.

**Table 8:** Summary of studies using microfluidic-based cultures in cancer research

Microfluidic device fabrication method	Caner type/cell line(s)	Objective(s)	Findings	References
Standard photolithography	Breast cancer/ MDA-MB-231	To create a matrix stimulating the TME.	- The model allowed for visualization of cell migration and cancer progression within the microenvironment. - The evolution of cell–cell interactions was time-dependent and thus can resemble in vivo activity.	[84]
Standard photolithography	Lung cancer/ SPCA-1and HFL1	To develop 3D cell culture in a microfluidic device that would allow for parallel testing of different chemotherapeutics.	- The 3D model accurately mimicked the TME and provided an efficient drug sensitivity testing platform. - Drug sensitivity behavior in 2D models differed significantly from that observed in the 3D model.	[85]
Soft photolithography	Breast cancer/ MCF-7	To establish a 3D model using hydrogel scaffolding in microwells, and to evaluate therapeutic effectiveness, distribution, and penetration of doxorubicin in the 3D cell culture.	- The microfluidic chip simulated the in vivo TME by providing a dynamic culture condition (i.e., fluid velocity, interstitial pressure). - 3D spheroids showed less sensitivity to doxorubicin compared to the 2D monolayers.	[146]
Low-pressure plasma oxidation	Lung cancer/ H292	To evaluate the therapeutic index of anti-EGFR-antibody cetuximab using human-skin co-culture assay.	- The integration of a metastatic with a scaled-down model of a functional human skin created an optimal test platform for assessing the effectiveness of EGFR inhibitors and other promising treatments in the field of oncology.	[147]
Multilayer photolithography	High-grade ovarian cancer/ OVCAR-8, FTSEC	To produce a custom-designed microfluidic device for the isolation of exosomes from patient serum samples and cultured cells.	- The microscale can facilitate the identification and isolation of exosome-derived biomarkers, which TME can be utilized in assays for the early detection of high-grade serous ovarian cancer.	[148]

## Challenges and future prospectives

While 3D cell culture offers many advantages over traditional 2D culture, it also presents some unique challenges that must be addressed to realize its potential for advancing research fully. One significant challenge is maintaining a stable and reproducible culture system. 3D cell culture systems often require specialized equipment, such as bioreactors and microfluidic devices, which can be expensive and difficult to use. These systems can be more challenging to reproduce compared to 2D systems due to the increased complexity and high heterogeneity of the culture environment, as cells are often embedded in matrices or scaffolds, making it difficult to control factors such as temperature, pH, and the presence of growth factors and/or other signaling molecules [149]. In addition, there is often a high degree of variability between different batches of cells and between experiments, making it difficult to draw statistically supported conclusions. Considering 3D cell cultures, adhering to Good Manufacturing Practices (GMP) principles is essential for translating these advanced models from research to clinical and commercial applications. However, several challenges and considerations arise when implementing GMP standards, including standardization of culture conditions, scalability, quality control, raw materials and biologics sourcing, regulatory compliance, data integrity, and documentation. GMP-compliant manufacturing processes require high reproducibility and control over critical parameters such as cell sourcing, culture media, culture supplements, and environmental conditions [150, 151]. As mentioned above, achieving this consistency can be challenging, given the inherent biological variability of primary cells and the sensitivity of 3D cultures to slight changes in culture conditions. Furthermore, meeting regulatory requirements is a paramount challenge in translating 3D spheroid cultures to clinical applications. Regulatory bodies, such as the Food and Drug Administration (FDA) in the United States and the European Medicines Agency (EMA) in Europe, have specific guidelines for the use of cell-based therapies and products [152]. GMP compliance is necessary to navigate these regulatory pathways and obtain approval for clinical trials and commercialization.

Moreover, oxygen accessibility is a critical consideration in 3D cell culture methods, and its heterogeneity within these environments poses a significant challenge in replicating physiological conditions and obtaining accurate experimental results. Cells located in the interior of 3D structures, such as spheroids, often encounter limited oxygen availability due to microenvironmental factors (i.e., tumor spheroids naturally develop hypoxic regions due to irregular vascularization in tumors) and diffusion barriers (e.g., densely packed cells, ECM, scaffolding matrices) [153]. As cells proliferate and form 3D structures, the demand for oxygen increases due to the larger volume that oxygen must traverse. Oxygen diffusion from the surrounding culture medium becomes progressively hindered as the distance from the culture surface to the interior of the 3D structure increases. This results in an oxygen gradient, where cells near the periphery have sufficient oxygen, but those in the core encounter oxygen deficiency, leading to hypoxia. Hypoxic core cells often exhibit altered gene expression, reduced proliferation, and changes in metabolic pathways as they enter a dormant state and cease cycling when deprived of oxygen and nutrients. This reduced activity renders them relatively resistant to cytostatic drugs that predominantly target actively dividing cells, leading to increased drug resistance, as is often observed in solid tumors [154, 155]. Confocal microscopy can be used to visualize dormant cells by labeling them with a nucleoside analog, allowing for their quantification and distinction from actively proliferating cells. This analog gets diluted in actively dividing cells. Still, it remains retained in quiescent, non-dividing cancer cells, thus providing a valuable tool for distinguishing them from the surrounding actively proliferating cells [156]. Leveraging this characteristic of 3D spheroids, they offer potential avenues for developing novel therapeutics targeting cancer cells resistant to cytostatic anticancer drugs. Wenzel et al. [157] cultivated T47D breast cancer cells in 3D cultures and used confocal imaging to differentiate cells within the inner core from those in the surrounding outer core. Cells in the inner core, experiencing limited access to oxygen and nutrients, exhibited reduced metabolic activity compared to their counterparts in the outer core. Through screening small molecule libraries against these 3D cultures, the authors identified nine compounds that selectively targeted and killed the inner core cancer cells while sparing the more actively proliferating outer cells. The identified drugs primarily affected the respiratory chain pathway, aligning with the altered metabolic activity of oxygen-deprived cells transitioning from aerobic to anaerobic metabolism. Hence, compounds selectively targeting dormant cancer cells significantly improved the effectiveness of commonly employed cytostatic anticancer drugs. Alternatively, the use of microfluidic devices that enable the creation of controlled oxygen gradients within cultures, the incorporation of oxygen-permeable materials, and the addition of oxygen-releasing compounds to provide a more uniform distribution of oxygen in vitro. However, it is important to acknowledge that these strategies may not fully replicate the complexity of oxygen gradients in real tissues [158]. Boyce et al. [159] presented the design and characterization of a modular device that capitalized on the gas-permeable properties of silicone to create oxygen gradients within cell-containing regions. The microfabricated device was constructed by stacking laser-cut acrylic and silicone rubber sheets, where the silicone not only facilitated oxygen gradient formation but also served as a barrier, separating the flowing gases from the cell culture medium to prevent evaporation or bubble formation during extended incubation periods. The acrylic components provided structural stability, ensuring a sterile culture environment. Using oxygen-sensing films, gradients with varying ranges and steepness in the microdevice can be achieved by adjusting the composition of gases flowing through the silicone elements. Furthermore, a cell-based reporter assay illustrated that cellular responses to hypoxia were directly proportional to the oxygen tension established within the system, proving efficacy.

Another practical challenge in 3D cultures arises from the intricacy of extracting cells from biomaterial-based 3D constructs. Typically, the construction of degradable hydrogel scaffolds involves integrating breakable crosslinks and/or cleavable components into the polymer structure or incorporating naturally biodegradable ECM constituents such as hyaluronic acid, laminin, fibronectin, and collagen [160]. Yet, traditional dissociation techniques prove to be notably inefficient and are influenced by the inherent structural complexities of the culture system. Enzymatic degradation, for example by collagenase, is a widely employed method for retrieving cells from 3D cell culture collagen-based scaffolds. The enzyme is selected to match the specific collagen type in the scaffold. During incubation, collagenase enzymatically cleaves the collagen fibers, releasing cells that were embedded or adhered to these fibers. Once the collagen has been broken down, the cells are collected as a suspension in the culture medium [161]. Cell viability and functionality assessments are typically performed to maintain the cells’ health and functionality. While using enzymatic degradation for 3D cell culture scaffolds is common, it remains an intricate approach associated with several limitations. It is important not to underestimate the impact of collagenase or other enzymes on cell viability and functionality. Careful optimization of digestion time and enzyme concentration is essential to balance efficient scaffold degradation and preserving cell quality [162]. Additionally, potential changes in cell phenotype during digestion are a significant concern, necessitating diligent monitoring of digestion parameters. In complex 3D scaffolds, particularly those with intricate structures, enzymatic digestion may be less effective, prompting researchers to explore alternative retrieval methods or adapt the digestion process. Ethical considerations also come into play, especially when working with human or animal-derived cells, raising concerns about using enzymes like collagenase. Adherence to ethical guidelines and institutional regulations is crucial for maintaining responsible and ethical research practices.

Hence, extensive research efforts have been directed toward developing improved techniques for cell retrieval from scaffold-based 3D cell cultures without compromising the cells’ integrity. For instance, Kyykallio et al. [163] developed an innovative pipeline for extracting extracellular vesicles (EVs) from 3D cancer spheroids using nanofibrillar cellulose (NFC) scaffolds as a cell culture matrix. This pipeline encompassed two distinct approaches: a batch method optimized for maximal EV yield at the conclusion of the culture period, and a harvesting method designed to facilitate time-dependent EV collection, allowing integration of EV profiling with spheroid development. Both approaches provided convenient setup, quick execution, and reliably produced a significant number of electric vehicles (EVs). Compared to scaffold-free 3D spheroid cultures on ultra-low affinity plates, the NFC-based approach demonstrated similar EV production per cell, offering scalability, preserved cell phenotype and integrity, and greater operational simplicity, ultimately leading to higher EV yields. Another approach is based on cell-mediated degradation of hydrogel scaffolds, where living cells actively break down the hydrogel structure [164]. This degradation mechanism is particularly relevant in tissue engineering and regenerative medicine. When cells are encapsulated within a hydrogel scaffold, they can secrete enzymes and other molecules that interact with its components, leading to its gradual breakdown. As cells proliferate and remodel their microenvironment, they may alter the scaffold’s properties and eventually facilitate its degradation. This dynamic process allows for the controlled release of cells, growth factors, and other bioactive substances within the hydrogel, making it a valuable technique for drug delivery applications.

While synthetic degradable polymer scaffolds are significant for developing 3D cell culture models, a concern regarding their in vitro and in vivo biocompatibility pertains to the presence of potentially toxic elements and chemicals utilized during the polymerization of synthetic hydrogels or the crosslinking of natural polymer hydrogel precursors, especially when the reaction conversion is less than 100%. These substances release unreacted monomers, stabilizers, initiators, organic solvents, and emulsifiers. These are integral to the hydrogel preparation process but may pose harm if they seep into the seeded cells or tissues [165, 166]. For instance, widely employed free radical photo-initiators (e.g., Irgacure) have been observed to diminish cell viability, even at minimal concentrations [167, 168]. Consequently, hydrogel scaffolds intended for embedding cells in 3D cultures typically require purification (e.g., by dialysis or solvent washing) to eliminate any residual hazardous chemicals before seeding. However, in certain scenarios, the purification of hydrogel scaffolds is more challenging or unfeasible, particularly when dealing with hydrogels generated through in situ gelation. In such cases, cells are introduced to the reactants necessary for hydrogel synthesis while still in a pre-polymer solution. As a result, when employing in situ gelation techniques, utmost caution must be exercised to ensure that all components are non-toxic and safe.

Furthermore, another challenge associated with 3D cell culture is the difficulty characterizing the cellular response to drugs and other therapeutic agents. In 2D cell culture, cells are typically analyzed using a range of standard assays that are well-established and easy to interpret. However, in 3D cell culture, there is often a lack of such standardized assays and protocols. Fang and Eglen [169] highlighted that the cultures’ complex morphology, functionality, and architecture hampered the application of some well-developed biochemical assays to 3D systems. Cells tend to aggregate into dense and/or large clusters over time, even in macroporous scaffolds, causing diffusional limitations when carrying out in situ characterization assays. Limitations arise due to the impeded diffusion and confinement of gases, nutrients, waste, and reagents within the system, compounded by challenges when quantifying and normalizing data between different biomimetic cultures [170–172]. For instance, Totti et al. [173] demonstrated that assessing a culture of pancreatic cancer cells in macroporous polyurethane foam-type scaffolds with the 3-(4,5-dimethylthiazol-2-yl)-5-(3-carboxymethoxyphenyl)-2-(4-sulfophenyl)-2H-tetrazolium (MTS) assay showed minimal differences between various scaffold conditions (e.g., ECM coatings on the scaffolds). However, sectioning, immunostaining, and imaging revealed clearer cell proliferation, morphology, and growth distinctions between the conditions. Likewise, the 3-(4,5-dimethylthiazol-2-yl)-2,5-diphenyl-2H-tetrazolium bromide (MTT) assay failed in capturing the differences in pancreatic cells’ viability cultured in polyurethane scaffolds after drug and irradiation screening, which were realized using advanced microscopy and imaging [174]. Hence, it is crucial for researchers to carefully consider the appropriate analytical approach that aligns with their study objectives before commencing the analysis of any 3D cultures. Also, they must be aware that some of the classical gold-standard approaches used in 2D cultures may not be directly applicable in 3D settings, as Hamdi et al. [175] showed that it is unfeasible to extract cells from spheroids for colony formation assays, which are used for developing post-treatment survival curves. Consequently, the researchers suggested in situ characterization readouts, which are novel and/or different from the existing 2D culture protocols.

Using stem cells and differentiated markers is crucial for characterizing and monitoring the cellular composition and differentiation status within 3D spheroids. These markers can help researchers achieve specific goals and outcomes, such as assessing the differentiation potential of stem cells, tracking the progression of differentiation, and studying the dynamics of cell populations in the spheroids [176, 177]. However, using such markers in 3D spheroid cultures presents certain challenges that need to be addressed for accurate and meaningful results. One primary challenge is the heterogeneity of stem cells within spheroids. Spheroids often comprise a mixture of stem cells and differentiated cells, so the stem cell markers may not exclusively identify and isolate the stem cell population, leading to difficulty in studying the specific behavior of stem cells within the spheroid. Another challenge is the variability in the expression of stem cell markers. These markers’ expression can fluctuate spatially and temporally within the spheroid, making it complex to track and interpret changes in marker expression over time. Additionally, in larger spheroids, stem cell markers may not effectively penetrate the core of the spheroid, limiting the ability to assess the stem cell population in the inner regions [176, 177]. Researchers can employ several strategies to overcome these challenges and effectively use stem cell markers in 3D spheroid cultures [178, 179]. An alternative method involves combining stem cells and other cellular markers to better understand the cellular composition within the spheroid. This multi-marker approach can help mitigate the issues related to marker heterogeneity. Moreover, live imaging techniques, such as confocal microscopy, can provide real-time insights into the dynamics of marker expression within spheroids. Controlling the size of spheroids is another strategy to enhance marker penetration and access to the innermost cells. Utilizing microfluidic techniques allows for the accurate regulation of spheroid size, ensuring effective penetration of markers throughout all regions of the spheroid [178, 179]. Additionally, single-cell analysis methods, such as single-cell RNA sequencing and proteomic analysis, enable the characterization of individual cells within spheroids. This approach can identify unique gene or protein expression patterns and shed light on the behavior of stem cell populations. Another valuable strategy is creating spheroids with genetically encoded stem cell reporters, which produce fluorescent or luminescent signals in stem cells, making them more visible and trackable. Lastly, mimicking the stem cell niche or microenvironment within 3D culture conditions can help maintain stemness and marker expression in spheroids [179].

Although imaging provides valuable information about cell distribution and binding, quantitative measurements using image analysis in 3D cultures are often lacking because they require cell count consistency across samples [180]. The challenge lies in the inability to visualize the whole-cell population, leading to difficulties obtaining accurate and reliable data from the entire culture. This is due to the hampered diffusion of fluorescent markers, primarily due to their large size, governed by the inherent heterogeneity of 3D cultures. One potential solution is to measure cell number from imageable cross-sections; however, Sirenko et al. [181] noted that light interferences and dye diffusion limitations resulted in unreliable results, as the number of cells counted substantially differed from the number of cells seeded. In addition, technical limitations such as prohibitive costs and limited scalability must also be considered [149]. Implementing 3D culture systems may incur higher costs compared to 2D culture systems, attributed to the requirement for specialized equipment, materials, and expertise [182, 183]. Similarly, scaling up 3D culture systems for industrial or clinical applications can be challenging due to the increased complexity of the culture environment and the need for specialized equipment [184]. This can limit the potential for the widespread adoption of 3D culture techniques in these settings.

Significant strides have been made in creating dynamic scaffolds that can respond to or guide resident cells [185]. For example, thermoresponsive hydrogels like poly-N-isopropylacrylamide (pNIPAm) have been proven effective for cell population harvesting [186, 187]. Moreover, the fusion of microscale technologies for cell culture with adaptable hydrogel designs has facilitated various investigations. These include investigating cell migration within microfluidic hydrogels and establishing high-throughput screening platforms to explore interactions between cells and materials [188]. Notably, the mechanobiology field is intrigued by various mechanically dynamic hydrogels that can either stiffen, soften, or reversibly transition between these states to examine cellular responses. These dynamic substrates offer a means to scrutinize how mechanical cues influence cell behavior, similar to the study of soluble factors over decades [189]. Techniques for introducing heterogeneity and multiple cell types within 3D constructs are also advancing. This includes innovative methods where hydrogels serve as bio-inks to print cells, either layer-by-layer from a 2D base or directly within a 3D space enclosed by another hydrogel. As these platforms progress, they are expected to become more widely accessible [190, 191]. In the interim, it remains crucial to maintain an open and collaborative dialogue between cell biologists, materials scientists, and engineers. This collaborative effort will ensure that the next generation of scaffold-based 3D cell culturing systems is well-equipped to address the significant challenges posed by the increasing biological and technical complexities.

## Conclusion

To conclude, scaffold-based 3D cell culture has emerged as a valuable tool in cancer research, providing a more physiologically relevant environment for studying tumor behavior, drug responses, and interactions between cancer cells and the surrounding microenvironment. Various scaffold materials, including polymers, decellularized tissue, hydrogels, and hybrids with microfluidics, have been explored to create complex and biomimetic 3D models. Polymer-based scaffolds offer tunable mechanical properties and are relatively easy to fabricate, making them versatile for 3D cell culture. The choice of polymers can influence cell behavior, proliferation, and migration, allowing researchers to study cancer progression and metastasis in a more realistic context. Additionally, incorporating bioactive molecules into polymer scaffolds can enable the controlled release of drugs and growth factors, facilitating drug screening and targeted therapy development. Furthermore, hydrogels offer high biocompatibility and can be functionalized with bioactive signals to direct cell behavior and tissue formation. In cancer research, hydrogels provide a platform to investigate the effect of mechanical cues on tumor growth, immune cell infiltration, and angiogenesis. Additionally, the ease of incorporating multiple cell types within hydrogels enables the study of tumor-stroma interactions. Likewise, decellularized tissue scaffolds retain native ECM composition, topography, and mechanical properties, closely mimicking the natural tumor microenvironment. As a result, cancer cells cultured in decellularized tissue scaffolds can exhibit more accurate tumor behaviors, including invasion and angiogenesis. Moreover, these scaffolds can be derived from patient-specific tissues, enabling personalized medicine approaches and improving the predictability of drug responses. Lastly, hybrid scaffolds that integrate microfluidic channels offer unique advantages for cancer research. By combining 3D cell culture with microfluidics, researchers can study tumor angiogenesis, metastasis, and drug penetration in a more physiologically relevant manner. Furthermore, microfluidics can facilitate high-throughput screening of anticancer drugs, enabling rapid and cost-effective testing of potential therapies.

## Data Availability

Not applicable.
